# Harnessing neutrophils for tissue repair and regeneration: mechanisms, biomaterial engineering strategies and translational potential

**DOI:** 10.1093/rb/rbag155

**Published:** 2026-07-21

**Authors:** Pengxiang Yang, Yuxiang Zhou, Xiaohang Zhang, Xiaomin Yao, Xingyu Jiang, Zizhen Gao, Leilei Gong, Yumin Yang, Jing Jie

**Affiliations:** Jiangsu Key Laboratory of Tissue Engineering and Neuroregeneration, Key Laboratory of Neuroregeneration of Ministry of Education, Co-innovation Center of Neuroregeneration, Nantong University, Nantong 226001, China; Department of Electrical Engineering, City University of Hong Kong, Hong Kong SAR 999077, China; Jiangsu Key Laboratory of Tissue Engineering and Neuroregeneration, Key Laboratory of Neuroregeneration of Ministry of Education, Co-innovation Center of Neuroregeneration, Nantong University, Nantong 226001, China; Jiangsu Key Laboratory of Tissue Engineering and Neuroregeneration, Key Laboratory of Neuroregeneration of Ministry of Education, Co-innovation Center of Neuroregeneration, Nantong University, Nantong 226001, China; Jiangsu Key Laboratory of Tissue Engineering and Neuroregeneration, Key Laboratory of Neuroregeneration of Ministry of Education, Co-innovation Center of Neuroregeneration, Nantong University, Nantong 226001, China; Jiangsu Key Laboratory of Tissue Engineering and Neuroregeneration, Key Laboratory of Neuroregeneration of Ministry of Education, Co-innovation Center of Neuroregeneration, Nantong University, Nantong 226001, China; Medical School of Nantong University, Nantong University, Nantong 226001, China; Jiangsu Key Laboratory of Tissue Engineering and Neuroregeneration, Key Laboratory of Neuroregeneration of Ministry of Education, Co-innovation Center of Neuroregeneration, Nantong University, Nantong 226001, China; Jiangsu Key Laboratory of Tissue Engineering and Neuroregeneration, Key Laboratory of Neuroregeneration of Ministry of Education, Co-innovation Center of Neuroregeneration, Nantong University, Nantong 226001, China; Jiangsu Key Laboratory of Tissue Engineering and Neuroregeneration, Key Laboratory of Neuroregeneration of Ministry of Education, Co-innovation Center of Neuroregeneration, Nantong University, Nantong 226001, China; Department of Clinical Laboratory, Southeast University Affiliated Nantong First People’s Hospital, Nantong 226001, China

**Keywords:** neutrophils, tissue repair, regeneration, immunomodulation, biomaterials

## Abstract

Tissue injury disrupts structural integrity and physiological homeostasis, triggering inflammatory and reparative responses that determine whether healing results in regeneration or fibrosis. Increasing evidence indicates that effective tissue restoration depends not only on the intrinsic regenerative potential of resident or transplanted cells but also on the spatiotemporal coordination of immune regulation, extracellular matrix remodeling and local microenvironmental signaling. Among immune populations, neutrophils are emerging as pivotal regulators of tissue repair rather than merely short-lived antimicrobial effectors. As the earliest leukocytes responding to injury, they rapidly sense inflammatory cues, traffic to injured tissues and shape the early regenerative niche through phagocytic clearance, reactive oxygen species-dependent and -independent effector functions, neutrophil extracellular trap formation and crosstalk with immune and structural cells. Their marked heterogeneity and phenotypic plasticity further expand their roles in determining repair outcomes. This review highlights how the distinctive biological properties of neutrophils are being translated into emerging engineering and therapeutic strategies, including neutrophil-inspired biomaterials, biomimetic delivery systems and immunomodulatory interventions. Current challenges in precise regulation, biosafety and clinical translation are also discussed. Collectively, neutrophils are positioned as both key immune regulators and promising bioinspired targets for next-generation regenerative medicine.

## Graphical Abstract

**Graphical Abstract:**
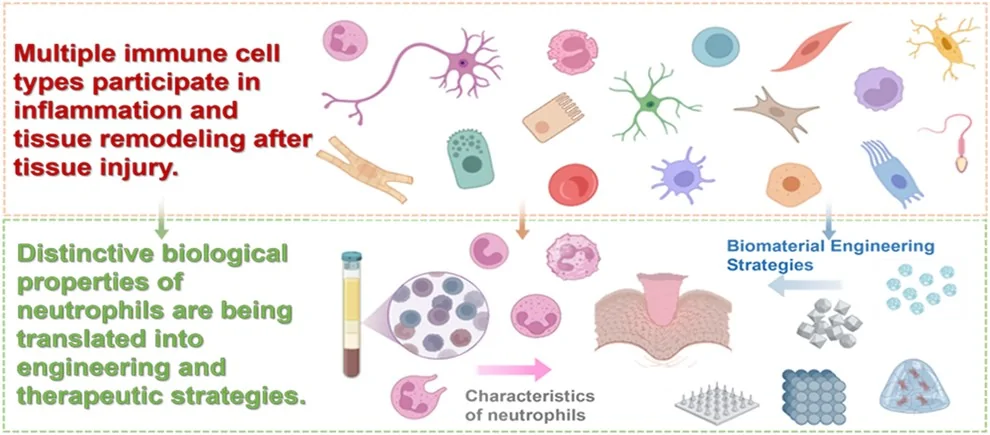

## Introduction

Biological tissues rely on highly ordered structure and homeostatic regulation to maintain their function. Various physical, chemical or pathological insults disrupt this balance and rapidly initiate a series of protective and reparative responses [1, 2]. Following tissue injury, the organism mounts multilayered adaptive responses in which inflammation, repair and regeneration constitute an integrated and dynamically coordinated continuum [3, 4]. These responses collectively form a complex regulatory network in which molecular signaling, immune cell behavior and extracellular matrix (ECM) remodeling are tightly coupled, ultimately determining whether structural restoration and functional recovery can be achieved [5, 6]. Across tissues including the nervous system, skin, vasculature and internal organs, the outcome of injury depends not only on intrinsic regenerative potential but also on whether the post-injury microenvironment provides appropriate spatiotemporal cues to guide cellular responses toward organized repair and reconstruction [7–9].

Despite possessing intrinsic reparative potential, tissues often rely on endogenous repair programs that, even when supported by progenitor activation and local regenerative signaling, remain insufficient to fully restore structure and function [10, 11]. Under many pathological conditions, persistent inflammation, aberrant ECM deposition and architectural disruption interfere with these regenerative programs, diverting healing toward scar formation rather than true regeneration [12, 13]. Such limitations are particularly evident in tissues with low regenerative potential, including the central nervous system, myocardium and long peripheral nerve defects. Even in tissues with moderate regenerative ability, aging, metabolic imbalance and chronic inflammation can disturb stem cell niches and disrupt the spatiotemporal coordination of repair signals, thereby weakening recovery [14–16]. Understanding why endogenous regenerative programs fail and which regulatory mechanisms determine the balance between regeneration and fibrotic repair, therefore, represents a central challenge in regenerative medicine.

Among the various regulatory systems involved, the immune system has emerged as a key determinant of injury outcomes, with neutrophils positioned at the forefront of this regulatory cascade [17, 18]. As the most abundant leukocyte population in peripheral blood, neutrophils have long been recognized as the first line of innate immune defense and their deficiency is associated with severe infections and high mortality, underscoring their indispensable role in host protection [19, 20]. Mature neutrophils originate from bone marrow hematopoietic stem cells and, despite their short lifespan, exhibit remarkable mobilization capacity, rapidly expanding and trafficking to sites of infection or injury. There, they exert potent defensive functions through phagocytosis, degranulation, reactive oxygen species production and neutrophil extracellular trap formation [21, 22]. However, these powerful effector mechanisms also carry the risk of collateral tissue damage, making the timely recruitment, activation and resolution of neutrophil responses critical for balancing host defense with tissue preservation.

**Figure 1:**
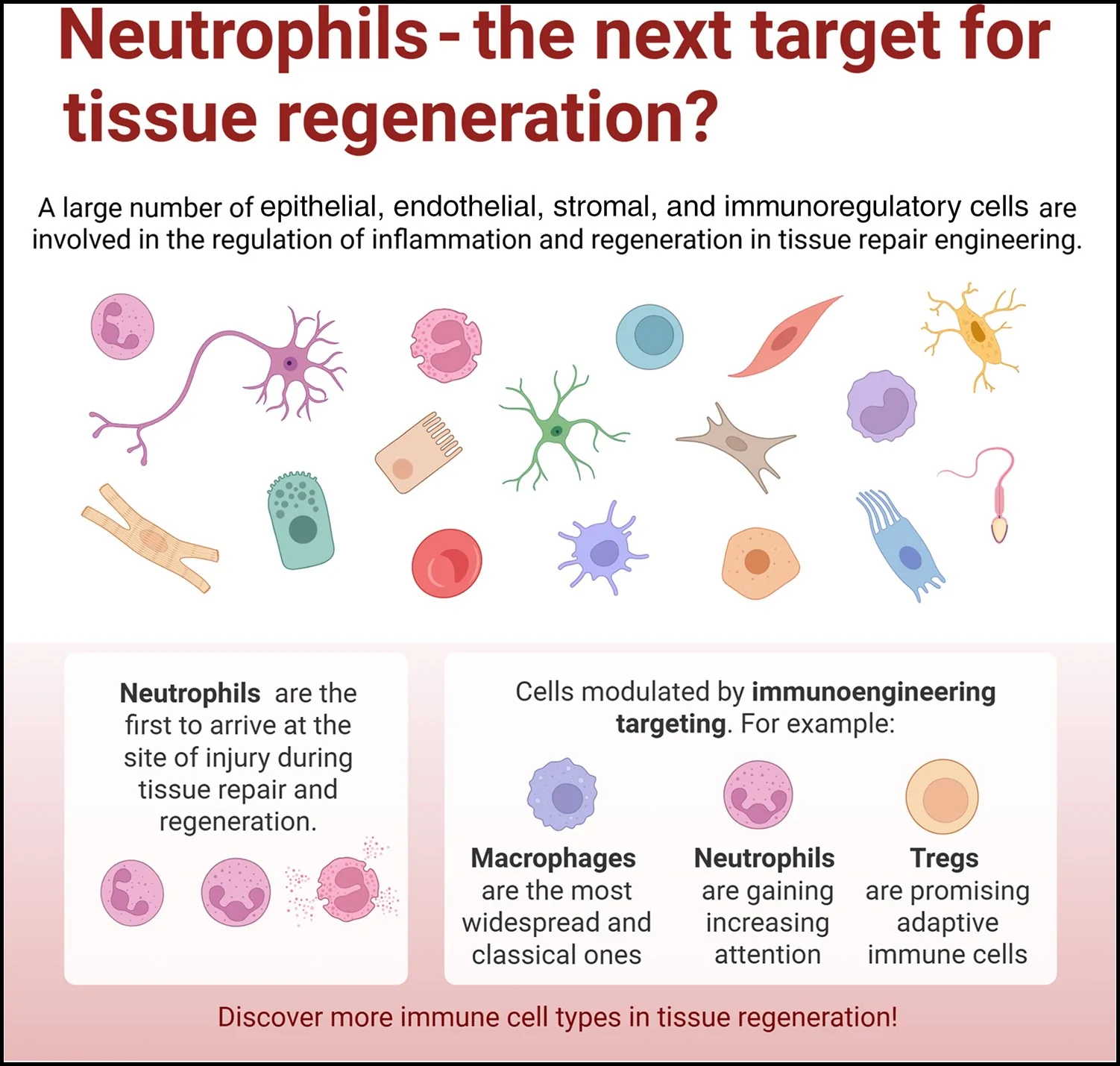
Neutrophils as emerging immunoregulatory targets in tissue repair and regeneration. Tissue repair is coordinated by dynamic interactions among structural cells and immune populations. As the first immune responders after injury, neutrophils critically shape the early repair microenvironment and are increasingly recognized as attractive targets for immunoengineering and regenerative therapy. Created with BioRender.com.

Recent advances have substantially expanded this traditional view, revealing that neutrophils are not merely inflammatory effectors but also active regulators of tissue repair [23]. Increasing evidence indicates that neutrophils contribute to immune modulation and microenvironmental remodeling during regeneration [24]. Single-cell and systems level analyses demonstrate pronounced phenotypic heterogeneity and functional plasticity, enabling neutrophils to influence macrophages, T cells and progenitor populations through cytokine secretion, chemotactic signaling and matrix-modifying factors, thereby shaping the transition from inflammation to regeneration [17, 22]. Notably, during the early phase of injury, neutrophils can participate in ECM remodeling and help establish provisional structural environments that support subsequent tissue reconstruction [7]. These findings reposition neutrophils as key coordinators linking immune responses to structural repair and highlight their emerging potential as therapeutic targets in regenerative medicine and immune engineering (Figure 1). In this review, we systematically summarize the structural features, effector mechanisms and regulatory roles of neutrophils in tissue repair and regeneration and discuss their theoretical and translational potential as central targets for immune-guided regenerative strategies.

## Structural and functional features of neutrophils as a basis for regenerative engineering

**Figure 2:**
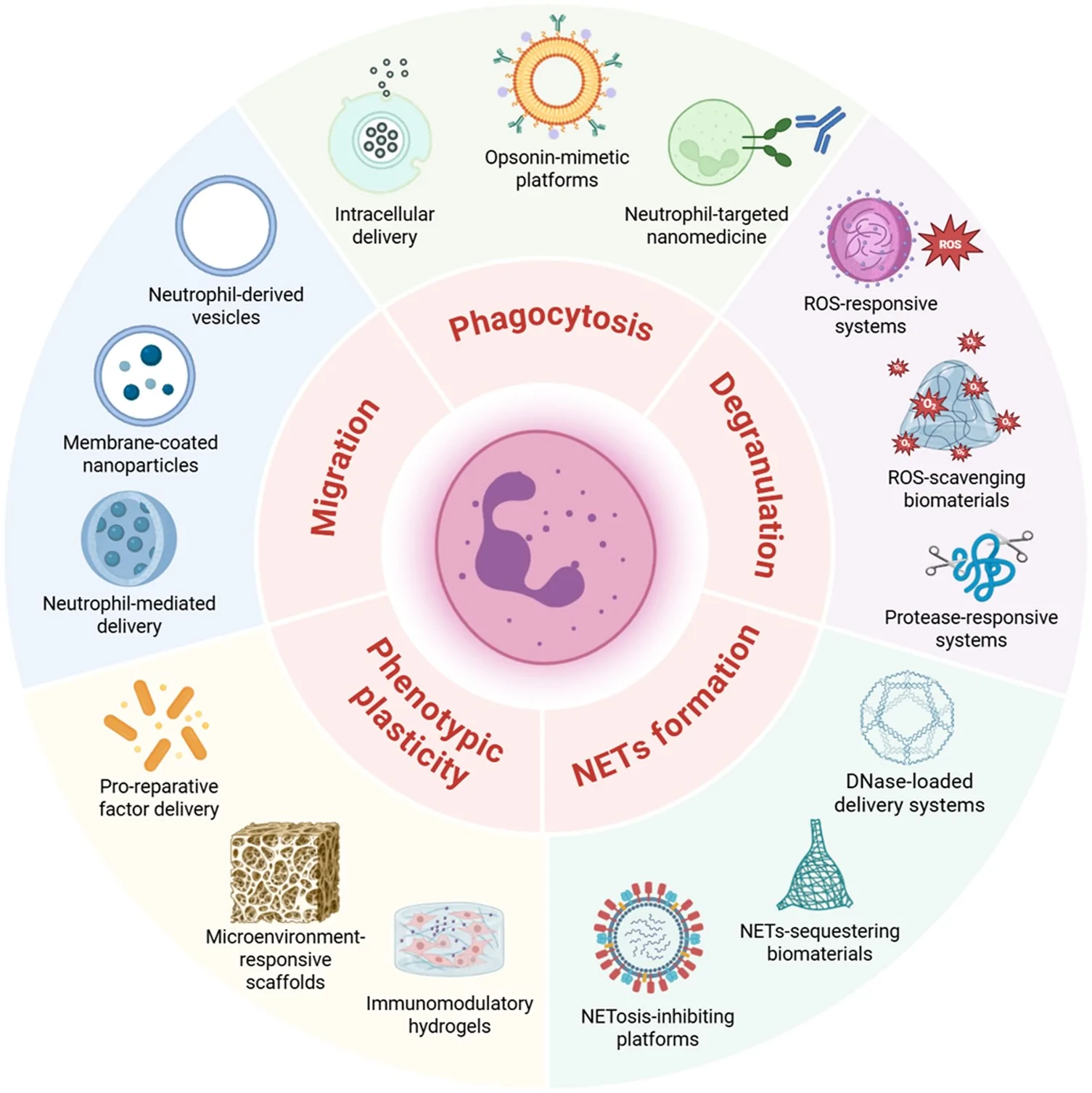
Biological functions of neutrophils and function-inspired biomimetic engineering strategies. Neutrophils exhibit multiple functional features, including migration, phagocytosis, degranulation, NET formation and phenotypic plasticity, which collectively contribute to inflammatory amplification, pathogen clearance and tissue repair. Based on these biological behaviors, engineered materials can regulate neutrophil-related responses through biomimetic delivery, targeted delivery, stimulus-responsive systems, ROS modulation, NET intervention and immune microenvironment remodeling. These strategies can not only suppress excessive inflammation and tissue damage but also promote the transition of neutrophils toward functional states that are more favorable for repair. Created with BioRender.com.

Neutrophils are the earliest immune cells recruited after tissue injury and therefore play a central role in shaping the initial inflammatory and repair microenvironment [25]. Their significance in regenerative medicine is not limited to rapid host defense. It also includes necrotic debris clearance, inflammatory resolution, ECM remodeling, regulation of vascular responses and interactions with macrophages, stromal cells, endothelial cells and progenitor cells. These activities make neutrophils important regulators of tissue repair and challenging targets for immune-guided regenerative engineering. This section discusses neutrophil features that are directly relevant to regenerative engineering. These include developmental origin, lifespan, rapid mobilization, granule release, ROS production, NET formation, functional plasticity and stage-dependent roles during tissue repair. On this basis, we further propose a classification framework for neutrophil-related biomaterial strategies (Figure 2).

### Developmental and structural basis of neutrophil function

Neutrophils originate from hematopoietic stem cell (HSCs) and progenitor cells in the bone marrow and undergo stepwise myeloid differentiation before entering the circulation as mature polymorphonuclear leukocytes [26–28]. This hematopoietic differentiation process is tightly regulated by multiple cytokines. Granulocyte colony-stimulating factor (G-CSF) serves as the key driver of neutrophil proliferation and maturation, while granulocyte–macrophage colony-stimulating factor (GM-CSF) promotes the generation of both granulocytes and monocytes. In addition, IL-3 and IL-6 act synergistically to support myeloid lineage development [29, 30]. Most mature neutrophils are temporarily stored in a bone marrow reserve pool, where they are enriched with cytoplasmic granules containing antimicrobial effector molecules, accounting for approximately 90% of the total population. The remaining 2–5% circulates in the peripheral blood and can be rapidly mobilized to tissues as needed to participate in immune defense [31–33].

**Figure 3:**
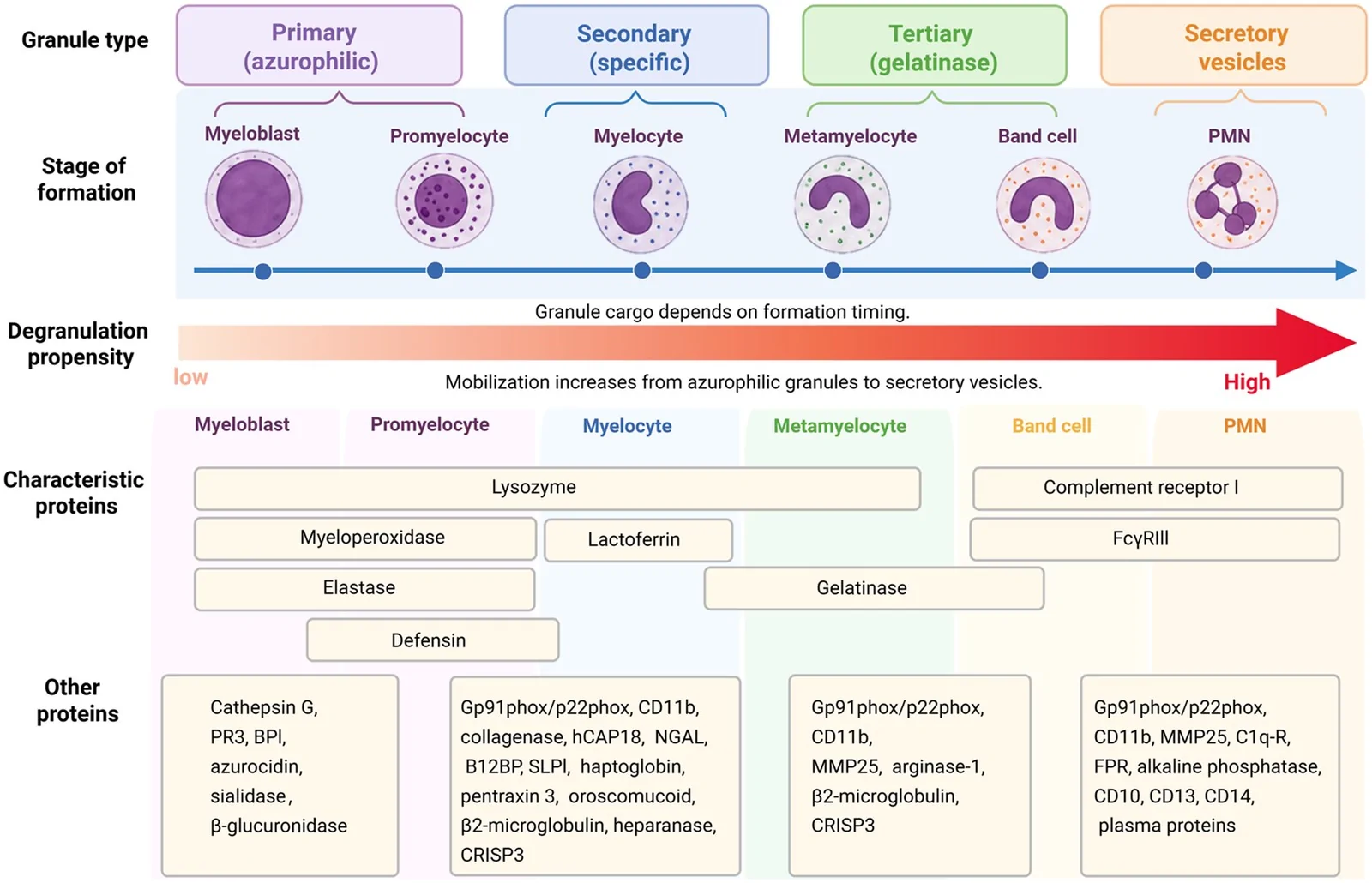
Neutrophil granules. Neutrophil granules contain antimicrobial proteins, enzymes, receptors and signaling molecules that support migration, host defense and inflammatory regulation. They are commonly classified as azurophilic, specific and gelatinase granules, with secretory vesicles also considered a related mobilizable compartment. These granule subsets differ in their stage of formation, molecular cargo and mobilization propensity, thereby enabling sequential release of effector molecules during neutrophil activation. Created with BioRender.com.

Several structural features of neutrophils are closely related to tissue repair and biomaterial engineering [34, 35]. Their deformable multilobed nuclei help them pass through narrow vascular and tissue spaces, while their granule-rich cytoplasm enables the rapid release of antimicrobial proteins, proteases, matrix-modifying enzymes and immunoregulatory mediators [36–38]. Granules are usually classified into azurophilic granules, specific granules and gelatinase granules, while secretory vesicles represent a highly mobilizable storage compartment [39–41]. The detailed classification and composition of neutrophil granules are shown in Figure 3 [42–44]. These granule subsets differ in molecular cargo and mobilization tendency, enabling neutrophils to release effector molecules in a relatively ordered manner during activation.

This staged release system is closely related to both repair and injury. Controlled granule release may support pathogen clearance, debris removal, inflammatory signaling and ECM remodeling. However, excessive or prolonged degranulation can damage surrounding tissues, degrade newly formed matrix and amplify inflammation [45, 46]. Thus, neutrophil granule content and release dynamics provide a biological basis for engineering strategies that mimic neutrophil functions, regulate endogenous neutrophil activation or respond to neutrophil-derived enzymes at injury sites [47–49].

In addition, secretory vesicles are often considered part of the neutrophil granule family. Unlike classical granules, they are not generated by budding from the Golgi apparatus but rather arise during late stages of neutrophil maturation through endocytosis [50, 51]. Their luminal content mainly consists of plasma-derived proteins such as albumin, whereas their membranes are enriched with adhesion molecules and receptors critical for neutrophil rolling, adhesion and transendothelial migration (TEM) [27, 52]. Granule subsets differ significantly in their mobilization capacity: secretory vesicles fuse with the plasma membrane most readily, followed by specific and gelatinase granules, whereas azurophilic granules show the lowest tendency to undergo exocytosis [53, 54]. This sequential mobilization, ordered according to the stage of formation, enables neutrophils to release effector molecules in a precise and timely manner during distinct phases of immune responses, thereby ensuring efficient execution of antimicrobial defense and immunoregulatory functions.

### Stage-dependent neutrophil functions and balanced regulation during tissue repair

Neutrophil functions during tissue repair are closely related to the stage of injury and the local microenvironment. After tissue injury, neutrophils are rapidly guided to the damaged site by chemokine gradients, endothelial adhesion signals and damage-associated molecular patterns (DAMPs). Once they enter the injured tissue, they participate in phagocytosis, degranulation, protease release, ROS production, NET formation, cytokine and chemokine signaling and extracellular vesicle (EV) release [4, 55]. These functions are essential for host defense and early tissue clearance, but their effects on regeneration depend on timing, response intensity, tissue context and the efficiency of inflammatory resolution.

In the early inflammatory phase, neutrophils mainly contribute to pathogen control, necrotic debris clearance and initiation of the repair response. Phagocytosis removes pathogens, dead cells and damaged matrix fragments, thereby preparing the injured site for subsequent repair. Controlled degranulation and protease release can facilitate cell infiltration and ECM remodeling, while ROS production contributes to antimicrobial defense and redox-dependent signaling [56]. Early NET formation may also help restrict pathogens and stabilize the local injury microenvironment [57]. However, these same effector mechanisms can become harmful when excessive or prolonged. Uncontrolled protease release may disrupt tissue architecture and degrade reparative matrix, whereas excessive ROS accumulation can induce oxidative injury, endothelial dysfunction, mitochondrial damage and persistent inflammation.

As the injury response progresses, neutrophils also participate in inflammatory resolution and microenvironmental remodeling. Neutrophil apoptosis, efferocytosis and reverse migration help limit local neutrophil accumulation and reduce secondary tissue injury. Clearance of apoptotic neutrophils by macrophages can promote the transition toward reparative macrophage phenotypes, thereby linking neutrophil clearance with immune resolution and tissue remodeling [58]. In this stage, neutrophils are not only inflammatory effector cells but also regulators of immune-cell crosstalk and the transition from inflammation to repair.

In the repair-promoting phase, neutrophil-derived cytokines, chemokines, lipid mediators, proteases and EVs may influence angiogenesis, ECM reorganization, epithelial or stromal cell behavior and progenitor-cell activity. These effects can support tissue reconstruction when they are spatially and temporally controlled [59, 60]. In contrast, persistent neutrophil activation may prolong inflammation and impair regeneration. Sustained NET formation or insufficient NET clearance may amplify inflammation, induce cytotoxicity, promote microvascular thrombosis, aggravate fibrosis and delay tissue repair.

Therefore, neutrophil functions should not be regarded as simply beneficial or detrimental. Their roles shift with the injury stage, activation level and local microenvironment. A central challenge in neutrophil-targeted regenerative therapy is how to preserve the beneficial functions of early neutrophil responses while preventing excessive or persistent tissue injury. Broad suppression of neutrophils is not an ideal strategy, because early neutrophil recruitment and activation are required for host defense, necrotic debris clearance and repair initiation. A more rational approach is to regulate the timing, intensity, localization and resolution of neutrophil responses. In this context, transient and localized neutrophil activity may support repair, whereas sustained or uncontrolled activation may become detrimental.

### Engineering implications and classification of neutrophil-related biomaterial strategies

The stage-dependent and environmental-dependent roles of neutrophils in tissue repair provide important design principles for regenerative engineering. Biomaterials can be used to spatially and temporally regulate neutrophil recruitment, activation, effector release and inflammatory resolution. For example, chemokine-presenting or chemokine-sequestering matrices may regulate the extent of neutrophil recruitment. ROS-responsive or antioxidant materials may limit oxidative damage while preserving necessary redox signaling. Protease-sensitive hydrogels may respond to neutrophil activity and release therapeutic cues in a feedback-dependent manner. Materials incorporating pro-resolving signals may further promote the transition from inflammation to repair. These strategies are intended to guide neutrophil activity toward localized, controllable and repair responses, rather than simply enhancing or suppressing neutrophil function.

At the same time, neutrophil-related engineering strategies face intrinsic biological limitations. Mature neutrophils are terminally differentiated, short-lived (approximately 6–8 hours) and difficult to expand *ex vivo*. Their activation state is highly sensitive to environmental cues, which complicates standardized preparation, storage, functional preservation and quality control. To address this limitation, recent studies have attempted to improve their engineering potential by regulating cell death pathways or reconstructing hematopoietic microenvironments. Luo *et al.* proposed a combinatorial treatment strategy termed CLON-G, which integrates caspase inhibition, lysosomal membrane permeabilization-oxidant blockade, necroptosis suppression and G-CSF supplementation. By simultaneously targeting multiple cell death pathways, this strategy extended the *ex vivo* half-life of neutrophils from less than 1 day to more than 5 days, while partially preserving phagocytosis, bactericidal activity, chemotaxis and ROS production. It also improved the shelf life of granulocyte infusion products [3]. In addition, engineered three-dimensional (3D) culture systems provide new opportunities for neutrophil induction and functional maintenance. Gonzalez-Fernandez *et al.* developed an injectable biomimetic GelMA hydrogel system to mimic the bone marrow niche and encapsulate hematopoietic stem and progenitor cells. This system induced the *in situ* differentiation of hematopoietic stem and progenitor cells into phagocytically functional neutrophils at infection sites, suggesting that artificial microenvironments can be used for *in vivo* immune cell induction [61]. These studies indicate that modulation of survival signals, cell death pathways and engineered niches may partly overcome the intrinsic limitations of neutrophil lifespan and expansion capacity.

Beyond direct neutrophil extension or induction, engineered 3D scaffolds can also serve as platforms for endogenous immune modulation. By tuning pore architecture, crosslinking density, mechanical properties, degradation kinetics and environmental responsiveness, biomaterials can influence the recruitment and functional state of endogenous neutrophils at injury sites. This may help sustain necessary inflammatory responses while preventing their persistence or dysregulation, ultimately creating a more favorable microenvironment for tissue regeneration. This perspective also helps connect neutrophil biology with the materials science of engineered niches [62–64]. Artificial cells and neutrophil-derived biomimetic systems represent another engineering direction. Artificial cells are generally constructed through synthetic biology, materials science and nanotechnology. They can integrate synthetic membranes, vesicles and functional modules such as enzymes, nucleic acids or nanoparticles to reproduce part or all of the functions of natural cells [65–68]. Compared with native cells, artificial cells offer advantages in programmability, modularity and customization [69, 70]. Although the structural and functional complexity of neutrophils still limits the construction of fully artificial neutrophils, neutrophil membranes, secretory vesicles and neutrophil-derived vesicles have been used to construct engineered carriers with inflammatory targeting capacity [10, 71, 72]. Wang *et al.* engineered ‘nanodecoy’ particles by coating polydopamine with neutrophil membranes to modulate the microenvironment of neural injury and promote regeneration [73]. Gao *et al.* used an extrusion method to generate nanoscale neutrophil vesicles for ranibizumab delivery, thereby inhibiting endothelial cell activation and proliferation [74].

Based on these biological features and engineering principles, it is useful to distinguish three major types of neutrophil-related biomaterial strategies before discussing specific neutrophil functions and their engineering implications. The first category is neutrophil-inspired or neutrophil-mimetic biomaterials, which reproduce selected structural or functional features of neutrophils, such as membrane receptors, adhesion molecules, chemotactic responses, inflammatory homing or NET-like structures. The second category is neutrophil-modulating biomaterials, which regulate endogenous neutrophil behavior at injury sites, including recruitment, activation, ROS production, protease release, NET formation, apoptosis, efferocytosis and resolution-related crosstalk with macrophages or stromal cells. The third category is neutrophil-as-carrier strategies, which directly exploit intact neutrophils or neutrophil-associated trafficking pathways to deliver drugs, nanoparticles or therapeutic cargoes to inflamed or injured tissues. These categories are not mutually exclusive, but they provide a useful framework for interpreting the function-oriented engineering strategies discussed in the following sections. Table 1 summarizes the biological basis, representative approaches, design purpose, advantages, key limitations and translational considerations of these strategies.

**Table 1:** Classification of neutrophil-related biomaterial engineering strategies.

Strategy category	Biological basis	Engineering approach	Main purpose	Advantages	Limitations
Neutrophil-inspired biomaterials	Neutrophil membrane, receptors, adhesion molecules, chemotaxis, NET-like structures	Membrane-coated nanoparticles, neutrophil-derived vesicles, artificial neutrophil-like systems, NET-inspired matrices	Mimic selected neutrophil functions	Inflammation targeting, immune evasion, biomimetic regulation	Source variability, limited functional complexity, safety and scalability concerns
Neutrophil-modulating biomaterials	Endogenous neutrophil recruitment, activation, ROS, proteases, NETs, apoptosis, efferocytosis	Chemokine-regulating matrices, antioxidant materials, ROS-responsive systems, protease-sensitive hydrogels, pro-resolution cues	Regulate local neutrophil responses	Local control, tunable immune modulation, repair-oriented regulation	Risk of impaired host defense, context-dependent effects, difficult timing control
Neutrophil-as-carrier strategies	Neutrophil chemotaxis, endothelial adhesion, tissue infiltration, barrier crossing	Drug-loaded neutrophils, nanoparticle-hitchhiking, neutrophil-mediated delivery	Use neutrophils as therapeutic carriers	Strong inflammatory homing, barrier penetration, targeted delivery	Short lifespan, activation risk, cargo retention, donor variability, manufacturing complexity

## Harnessing neutrophil recruitment and trafficking for targeted repair

A defining feature of neutrophils in tissue injury is their rapid and highly regulated trafficking behavior. From chemotactic recruitment to TEM and reverse migration, these dynamic processes determine not only the timing and magnitude of neutrophil accumulation but also their functional impact on inflammation, repair and tissue homeostasis [75]. This section therefore focuses on the key mechanisms governing neutrophil recruitment and trafficking, including factor-driven chemotaxis, receptor-mediated TEM and reverse TEM, with particular emphasis on their therapeutic relevance and engineering potential.

### Recruitment cues and inflammation-targeting strategies

Upon tissue injury or infection, damaged cells release a variety of molecular signals, including DAMPs and pathogen-associated molecular patterns (PAMPs). These signals are recognized by pattern recognition receptors (PRRs) on innate immune cells, thereby activating downstream signaling cascades that induce transcription factors such as NF-κB and AP-1 to translocate into the nucleus [4, 76, 77]. This leads to the production of pro-inflammatory cytokines, including IL-1, IL-6 and TNF-α, by macrophages and neutrophils. As the inflammatory response is initiated, resident macrophages, endothelial cells and local tissue cells secrete abundant chemokines such as CXCL1, CXCL2, CXCL3, CXCL5, CXCL6 and CXCL8 (IL-8), establishing stable chemical gradients that guide the directional migration of neutrophils [2, 78, 79]. Among these mediators, IL-8 is considered the most potent neutrophil chemoattractant. By binding to CXCR1 and CXCR2 on neutrophils, IL-8 activates multiple downstream signaling pathways, including MAPK, PLC and PI3K, thereby driving neutrophil chemotaxis, activation and effector functions [6, 80, 81]. Beyond recruitment, IL-8 also interacts with endothelial cells, tumor cells and stem cells, contributing to angiogenesis, cellular proliferation and tissue repair [82, 83]. However, the concentration of IL-8 must be tightly regulated; insufficient levels fail to promote effective repair, whereas excessive levels can result in tissue injury and exacerbated inflammation. In addition, IL-33 is abundantly released during tissue injury as an ‘alarmin.’ By engaging its receptor ST2, IL-33 enhances neutrophil adhesion and recruitment while also activating local mast cells and T cells [84–86]. These cells, in turn, release TNF, IL-1 and IL-6 to augment endothelial adhesion capacity for neutrophils. Activated mast cells and T cells further secrete CXCL1, CXCL2 and IL-8, amplifying neutrophil recruitment in a positive feedback loop. This coordinated network ensures the rapid accumulation of neutrophils in the early stages of injury, providing the cellular foundation for necrotic tissue clearance, neovascularization and subsequent tissue repair.

**Figure 4:**
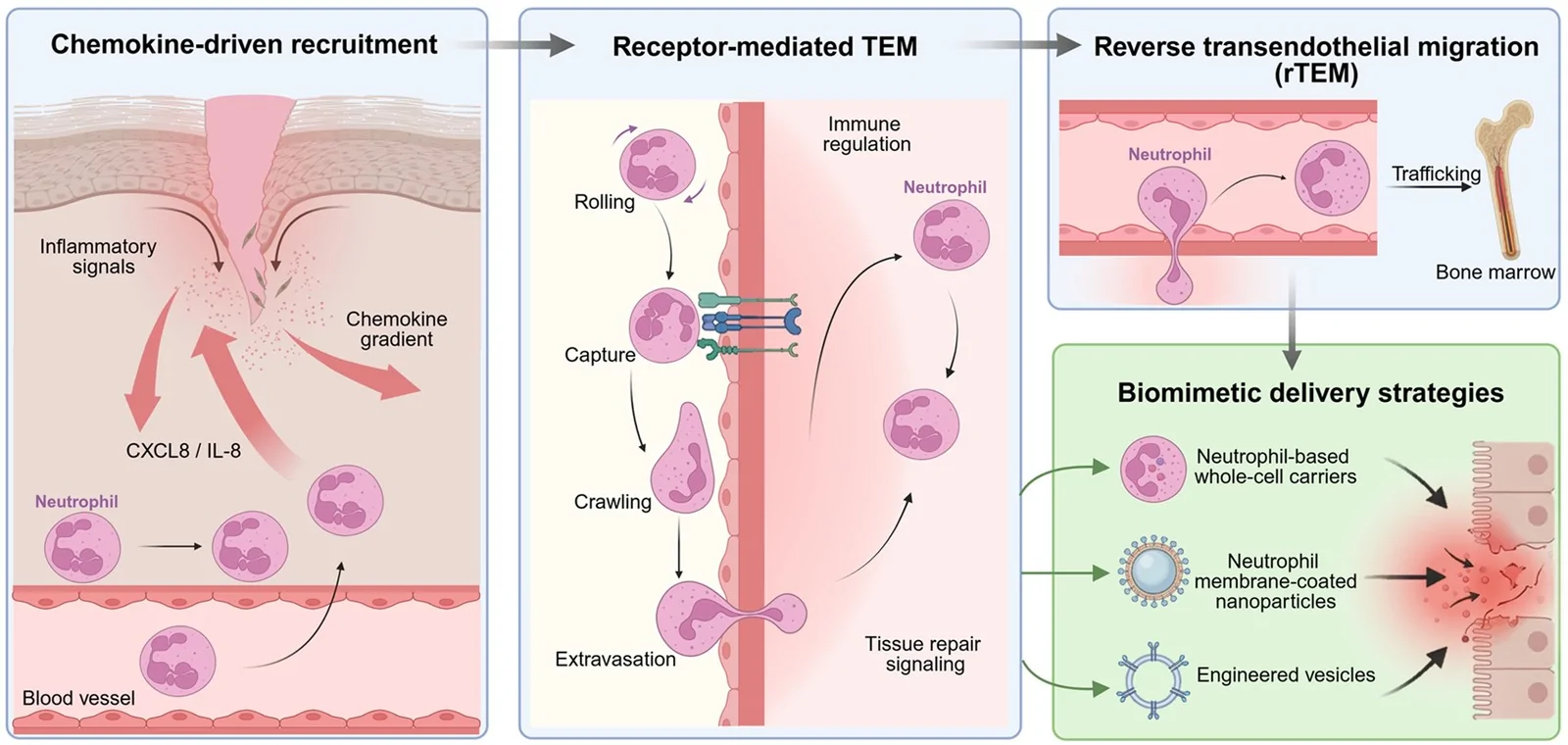
Migratory modes of neutrophils during tissue injury and biomimetic delivery strategies. After tissue injury, neutrophils are rapidly recruited by chemokine gradients, undergo TEM across activated vascular endothelium and may subsequently participate in reverse migration or recirculation. These trafficking behaviors determine the timing, localization and magnitude of neutrophil responses during inflammation and repair. They also provide biological inspiration for engineering strategies, including inflammation-targeted delivery, neutrophil membrane-coated nanoparticles, engineered vesicles and neutrophil-mediated cargo transport. TEM, transendothelial migration; rTEM, reverse transendothelial migration. Created with BioRender.com.

Building on the unique properties of neutrophils, Gao *et al.* developed an ophthalmic formulation based on neutrophil-derived nanovesicles. By exploiting chemokine receptors present on neutrophil membranes, this system actively homed to inflamed sites and achieved precise and efficient therapeutic effects [74]. In another study, Myriam *et al.* demonstrated that platelets facilitate early neutrophil recruitment to injured muscle tissue through the release of chemokines CXCL5 and CXCL7, thereby promoting muscle repair. Platelet depletion or genetic deletion of CXCL7 markedly reduced neutrophil infiltration, resulting in exacerbated inflammation, impaired myofiber growth, incomplete muscle functional recovery and defective neovascularization [87]. Beyond chemokines, tyrosine kinase Syk has been identified as a key regulator of neutrophil activation in the early stages of spinal cord injury (SCI). Pharmacological inhibition or neutrophil-specific knockout of Syk significantly alleviated neural tissue damage, restrained the spread of inflammation and effectively promoted long-term functional recovery [88]. Throughout the tissue repair process, neutrophil recruitment and activation exhibit a marked cascade amplification effect. An appropriate early neutrophil response helps clear necrotic tissue and pathogens and initiates subsequent repair processes. However, excessive recruitment or sustained activation may prolong inflammation, leading to tissue damage, chronic inflammation and even fibrosis. Therefore, recruitment-oriented engineering strategies should not simply aim to enhance neutrophil homing, but should instead emphasize precise control over recruitment intensity, timing and local specificity. From a materials design perspective, chemokine-guided neutrophil migration provides an important basis for inflammation-targeted delivery and local immune modulation. Materials capable of presenting, retaining or releasing chemokine-like cues can regulate neutrophil accumulation at injured sites, while neutrophil membrane-coated vesicles or nanoparticles can exploit chemokine receptor-mediated homing for targeted drug delivery. By controlling the spatial distribution and temporal window of neutrophil recruitment, these strategies may preserve the early repair-supportive effects of neutrophils while reducing persistent inflammation and secondary tissue injury (Figure 4) [23, 60].

### Transendothelial migration as a basis for biomimetic delivery

The TEM of neutrophils is a pivotal step in the inflammatory response, relying on the precise coordination of pro-inflammatory signals and receptor-ligand interactions. During inflammation, cytokines such as TNF-α, IL-1β and IL-17 activate vascular endothelial cells, inducing the high-level expression of adhesion molecules on the luminal surface, including P-selectin, E-selectin and intercellular adhesion molecules (ICAMs) [89, 90]. Circulating neutrophils continuously scan the vascular endothelium, particularly within post-capillary venules characterized by slower blood flow and narrower luminal diameters, where encounters with activated endothelial cells are more frequent. During transmigration, mechanical forces imposed at narrow endothelial junctions are sensed by the mechanoreceptor Piezo1 located on neutrophil membranes. This mechanosensory activation triggers transient intracellular Ca^2+^ signaling fluctuations and upregulates NADPH oxidase IV expression, thereby significantly enhancing neutrophil bactericidal activity [91].

Two constitutively expressed glycoproteins on the neutrophil surface, P-selectin glycoprotein ligand-1 (PSGL-1) and L-selectin, bind to P-selectins and E-selectins on endothelial cells, initiating the rolling behavior of neutrophils [6]. This initial step not only mediates deceleration and transient anchoring of neutrophils along the vascular wall but also activates intracellular signaling pathways, including Src family kinases, Syk, PI3K and p38 MAPK, which subsequently drive cytoskeletal remodeling and other downstream biological responses. In an innovative biomimetic approach, Shi *et al.* engineered bispecific multivalent peptides that mimic the structural features of PSGL-1 and simultaneously target P-selectins and E-selectins. When conjugated onto the surface of mesenchymal stem cells (MSCs), these peptides markedly enhanced MSC homing efficiency. Moreover, by competitively inhibiting excessive neutrophil infiltration, the engineered MSCs alleviated inflammatory damage, improved the immune microenvironment and promoted hepatic tissue repair. This dual-functional strategy provides a novel paradigm for simultaneously optimizing therapeutic cell homing and restraining aberrant neutrophil accumulation [13].

As signaling cascades progress, β2 integrins on the neutrophil surface, such as LFA-1 and Mac-1, become activated and clustered, enabling specific binding to ICAM-1 on endothelial cells. This transition marks the ‘firm adhesion’ phase, which halts rolling and firmly anchors neutrophils to the endothelium. Engagement of integrins with ICAMs not only stabilizes neutrophil attachment but also promotes the formation of leading-edge pseudopodia, preparing the cells for tissue entry. Guided by exogenous or endogenous chemotactic cues, neutrophils undergo further cytoskeletal rearrangements, migrate directionally along chemotactic gradients and initiate antimicrobial responses such as the respiratory burst, thereby completing TEM [92, 93]. Following passage through endothelial junctions, neutrophils must also breach the basement membrane, which is primarily composed of laminin and type IV collagen. Although the exact mechanisms remain incompletely defined, it is widely accepted that proteolytic enzymes, particularly MMPs, facilitate this process via degranulation-mediated proteolysis, enabling neutrophils to infiltrate damaged or infected tissues and exert their immune defense functions. Harnessing the intrinsic inflammation-homing property of neutrophil membranes to traverse barriers such as the blood-brain barrier (BBB) has further enabled efficient targeted delivery to ischemic stroke lesions [94].

The TEM process also provides useful principles for biomimetic delivery across vascular barriers. Adhesion molecules, selectin-ligand interactions, integrin activation and endothelial junction remodeling can inspire the design of cell-based carriers, membrane-coated nanoparticles or ligand-modified biomaterials with improved vascular homing and tissue penetration. Such strategies may be particularly relevant for inflamed tissues or barrier-protected lesions, where conventional delivery systems show limited access. Nevertheless, reproducing neutrophil-like TEM is challenging, because adhesion strength, endothelial activation, shear stress and tissue-specific vascular properties must be balanced. Uncontrolled imitation of neutrophil adhesion or transmigration may also increase vascular inflammation or off-target accumulation. Thus, TEM-inspired engineering should aim for selective vascular targeting and controlled barrier crossing rather than nonspecific enhancement of cell adhesion.

### Reverse migration and its translational potential

Reverse transendothelial migration (rTEM) refers to the process whereby neutrophils, after mounting an initial response at sites of inflammation or infection, do not remain within the interstitial tissue but instead reverse their traditional migratory route and re-enter the vascular system. This phenomenon stands in sharp contrast to classical TEM (from vasculature into tissues) and underscores the remarkable dynamism and plasticity of neutrophils in both physiological and pathological contexts [95, 96]. At the molecular level, rTEM depends on the precise engagement of neutrophil surface adhesion molecules, such as β2 integrins, with their endothelial ligands, and is further orchestrated by pro-inflammatory cytokines including TNF-α and IL-1β as well as chemokine gradients. Relaxation of endothelial junctions, remodeling of the basement membrane and the establishment of ‘reverse chemotactic gradients’ within the inflammatory microenvironment together provide the permissive conditions required for rTEM [97, 98]. This mechanism not only facilitates the recirculation of neutrophils after fulfilling their local effector functions but may also exert significant influence under specific pathological conditions.

rTEM carries important implications under both physiological and pathological conditions. Under physiological settings, rTEM contributes to neutrophil clearance and recirculation, thereby promoting the resolution of inflammation, limiting tissue damage and maintaining homeostasis [99]. In contrast, under pathological conditions such as systemic infection, tumor progression or autoimmune diseases, rTEM may serve as a conduit for the dissemination of inflammatory mediators, secondary tissue injury or aberrant immune activation [100]. Thus, rTEM can function either as a pivotal checkpoint for ‘inflammation resolution’ or, conversely, as a mechanism exacerbating disease progression in certain contexts. In a study published in Nature Nanotechnology, the group led by Professor You developed an innovative bone marrow-targeted drug delivery system based on the homing mechanism of senescent neutrophils. Exploiting the upregulation of CXCR4 during neutrophil aging, the system harnesses the natural CXCR4/CXCL12 axis to direct neutrophils toward the bone marrow in response to CXCL12 secreted by stromal cells. Free drugs or nanotherapeutics can thus be ‘hitchhiked’ on senescent neutrophils and efficiently delivered across the blood-bone marrow barrier. This approach demonstrated significant therapeutic benefits in models of breast cancer bone metastasis and osteoporosis. Notably, the system required no *ex vivo* cell engineering, instead leveraging innate immune cell pathways to achieve precise, safe and effective targeting, underscoring its strong potential for clinical translation [101, 102]. Although the mechanisms and functional consequences of rTEM remain incompletely understood, this process provides a useful framework for reconsidering neutrophil trafficking beyond unidirectional tissue entry. By involving neutrophil exit, recirculation and redistribution, rTEM may inspire neutrophil-associated engineering strategies, including hitchhiking delivery systems, inflammatory cargo transport and bone marrow- or circulation-directed targeting. It may also inform the design of materials that facilitate neutrophil clearance after the early inflammatory phase, thereby supporting inflammatory resolution. However, because reverse-migrated neutrophils may either promote resolution or disseminate inflammatory signals depending on the disease context, rTEM-related strategies remain exploratory and require clearer definition of neutrophil phenotype, activation state and destination after reverse migration.

Overall, neutrophil recruitment and trafficking provide a mechanistic basis for their rapid response to tissue injury and also offer practical entry points for biomaterial engineering. Chemokine-driven recruitment can guide inflammation-targeted delivery, receptor-mediated TEM can inspire vascular homing and barrier-crossing systems, and reverse migration may inform neutrophil-associated trafficking, redistribution and resolution-oriented strategies. At the same time, these approaches face common challenges, including the difficulty of controlling neutrophil activation intensity, recruitment duration, tissue specificity and off-target inflammatory effects. Therefore, engineering strategies based on neutrophil trafficking should aim to modulate neutrophil distribution and function with precise spatial and temporal control.

## Harnessing neutrophil effector functions to remodel the regenerative microenvironment

Beyond their rapid recruitment to injured tissues, neutrophils actively shape the regenerative microenvironment through a series of effector functions. Among these, phagocytic clearance of cellular debris, oxygen-dependent killing through respiratory burst and oxygen-independent antimicrobial and regulatory mechanisms represent three major functional dimensions that influence early repair and therapeutic intervention [22, 103]. This section summarizes these core neutrophil functions and the corresponding strategies developed on their basis, while neutrophil extracellular traps (NETs), owing to their distinct biological and translational significance, are discussed separately in the following chapter (Table 2) [104–127].

**Table 2:** Neutrophil effector functions in microenvironment remodeling and engineering applications.

Function	Mechanism	Representative molecules/components	Microenvironmental features	Representative engineering or therapeutic applications	Advantages	Limitations and translational concerns	References
Phagocytosis	Neutrophils recognize and internalize pathogens, apoptotic bodies and tissue debris through receptor-mediated engulfment. Recognition occurs either directly via PRRs sensing PAMPs/DAMPs or indirectly through opsonin-dependent pathways, including Fcγ receptor- and complement receptor-mediated phagocytosis. Phagosomes subsequently fuse with intracellular granules to acquire microbicidal activity.	PRRs, FcγRs, complement receptors, phagosomes, azurophilic granules, specific granules, lysozyme, MPO, NE, CG	Rapid particle internalization; direct fusion of phagosomes with granules; early neutral to mildly alkaline phagosomal milieu that supports protease activation and efficient intraphagosomal killing	Biomaterials and nanoplatforms engineered with immune-active ligands, antibody fragments, or ordered surface proteins to enhance neutrophil recognition and uptake; neutrophil-tropic nanoparticles for inflammatory site-targeted drug delivery	Supports pathogen and debris clearance; enables neutrophil-mediated uptake and inflammatory targeting; may improve early repair microenvironment	Excessive neutrophil activation may aggravate inflammation and tissue injury; premature phagocytic uptake may reduce circulation time of nanomedicines; difficult to control whether phagocytosis should be enhanced or avoided in different disease stages	[103–110]
Oxygen-dependent killing	Activation of the NADPH oxidase complex on phagosomal or plasma membranes initiates the reduction of molecular oxygen to superoxide, followed by ROS cascade formation, including hydrogen peroxide and hypochlorous acid. ROS interact with granule enzymes and ionic fluxes to create a highly toxic antimicrobial compartment.	NADPH oxidase complex, superoxide, H_2_O_2_, MPO, HOCl, ONOO^−^	ROS-rich and highly oxidative microenvironment; localized redox stress; coordination with ion flux and protease activation; neutral or weakly alkaline phagosomal conditions distinct from macrophages	Neutrophil membrane-coated nanoparticles, ROS-scavenging nanomaterials, ROS-responsive drug-release systems and catalytic neutrophil-mimetic or neutrophil-guided platforms designed to regulate oxidative microenvironments and improve targeted therapy	Enables antimicrobial defense; provides ROS-responsive triggers for local drug release; supports design of antioxidant or redox-regulating biomaterials	Excessive ROS cause oxidative injury, endothelial dysfunction, mitochondrial damage and persistent inflammation; broad ROS suppression may impair antimicrobial defense and redox-dependent repair signaling; therapeutic window is tissue- and stage-dependent	[111–118]
Non-oxidative antimicrobial activity	Neutrophils deploy granule-derived antimicrobial peptides, proteases and metal-chelating proteins to kill pathogens independently of ROS. These molecules disrupt microbial membranes, interfere with intracellular metabolism, degrade virulence factors and restrict nutrient availability through metal sequestration.	α-defensins, LL-37, lysozyme, NE, CG, PR3, lactoferrin, calprotectin	Protease-rich and antimicrobial protein-enriched niche; local deprivation of essential metal ions such as iron, zinc and manganese; maintains antimicrobial capacity under hypoxic or ROS-deficient conditions	Antimicrobial peptide-inspired biomaterials, protease-regulating delivery systems, granule protein-mimetic therapeutic strategies and materials designed to exploit nutritional immunity or reinforce local antimicrobial defense	Provides antimicrobial activity under hypoxic or ROS-limited conditions; offers molecular templates for antimicrobial and protease-responsive biomaterials; may reinforce local host defense	Excessive protease activity may degrade reparative ECM and damage host tissues; antimicrobial peptides or metal-chelating systems may cause cytotoxicity or disturb normal cell metabolism; difficult to balance antimicrobial potency with tissue compatibility	[119–126]

PRRs, pattern recognition receptors; PAMPs, pathogen-associated molecular patterns; DAMPs, damage-associated molecular patterns; FcγRs, Fc gamma receptors; MPO, myeloperoxidase; NE, neutrophil elastase; CG, cathepsin G; PR3, proteinase 3; ROS, reactive oxygen species.

### Phagocytic clearance in early repair

Phagocytosis is one of the principal mechanisms by which neutrophils eliminate pathogens and cellular debris. It is an active, receptor-mediated process whereby neutrophils recognize and internalize foreign particles through the plasma membrane, forming a membrane-bound vacuole known as the phagosome [104–106]. Interactions with microbes and damaged materials can occur through two major pathways: (i) direct recognition of PAMPs or DAMPs via PRRs and (ii) indirect recognition mediated by opsonins such as IgG and complement proteins. Among these, Fcγ receptor (FcγR)-mediated phagocytosis requires the formation of pseudopodia to engulf IgG-opsonized particles, whereas complement receptor-mediated phagocytosis often occurs without extensive membrane protrusion [107, 108]. These opsonin-dependent mechanisms have been more thoroughly elucidated in research, highlighting the flexibility and efficiency of neutrophils in mounting rapid and broad-spectrum phagocytic responses.

However, nascent phagosomes in neutrophils do not initially possess potent bactericidal activity; they acquire such lethal properties only after undergoing a subsequent maturation process [128–130]. This maturation differs significantly from that observed in macrophages. In macrophages, phagosomes typically progress along a classical maturation pathway through sequential fusion with endosomes and lysosomes [79, 131]. In contrast, neutrophil phagosomes directly fuse with multiple intracellular granules, including azurophilic and specific granules, thereby rapidly delivering a diverse array of antimicrobial molecules, such as lysozyme, MPO and elastase, into the phagosomal lumen. At the same time, NADPH oxidase assembles on the phagosomal membrane, driving a burst of ROS production that creates a highly oxidative and destructive microenvironment for pathogens. The synergy of these processes markedly enhances the bactericidal efficacy of neutrophil phagosomes [132–134]. pH regulation within neutrophil phagosomes also diverges from that of macrophages. While macrophage phagosomes progressively acidify during maturation, neutrophil phagosomes initially exhibit an alkaline pH and remain near neutral for a relatively extended period. This unique environment is crucial for the activation of neutrophil serine proteases such as NE and CG. Although acidification later occurs through the fusion of acidic granules, sustained NADPH oxidase activity drives proton extrusion, thereby maintaining phagosomal pH within an optimal range for the activity of bactericidal proteases [135–137].

Enhancing neutrophil phagocytosis is an important strategy to strengthen host defenses against infection and cancer. Studies have shown that specific cytokines, such as GM-CSF, G-CSF and IFN-γ, can markedly augment neutrophil activation and phagocytic capacity. In addition, the deposition of complement fragment C3b and IgG significantly improves the efficiency of target recognition by neutrophils [109–111]. In recent years, an increasing number of biomaterials and nanodrug delivery platforms have been designed to promote neutrophil phagocytosis. Strategies include surface modification with immune-active ligands or antibody fragments to direct neutrophils toward rapid binding and internalization, thereby facilitating pathogen clearance or targeted drug delivery [138–141]. These represent promising applications of bioinspired engineering approaches. Notably, the supramolecular arrangement of proteins on nanoparticle surfaces exerts a profound influence on their immune targeting properties, acting as a ‘hidden code’ that determines nanoparticle fate. For instance, nanoparticles with an ordered surface display of albumin acquire a selective ‘tropism’ for neutrophils, enabling preferential uptake during acute lung inflammation and achieving targeted delivery to inflamed sites [142].

Despite the establishment of a highly efficient bactericidal system, many pathogens have evolved diverse strategies to evade neutrophil-mediated immunity. Some express host-mimicking molecular signals, such as the ‘don’t eat me’ signal CD47, to suppress phagocytosis, whereas others secrete proteins that degrade complement components or antibodies, thereby reducing the efficiency of opsonization [143, 144]. Certain bacteria rely on antioxidant enzyme systems to counteract ROS-mediated killing within phagosomes, or exploit rapid motility and biofilm formation to create physical barriers that hinder effective neutrophil clearance [145]. Tumor cells employ similar tactics, expressing immune checkpoint molecules such as PD-L1 to facilitate immune evasion [146, 147]. In the field of nanomedicine, these principles have been ingeniously leveraged in the design of drug delivery systems. For example, nanoparticles modified with polyethylene glycol (PEG) or cloaked with natural cell membranes can effectively evade neutrophil phagocytosis, thereby prolonging circulation time and enhancing drug targeting [148, 149]. Elucidating these immune evasion mechanisms not only deepens our understanding of the immunological basis of infection and tissue repair but also provides a theoretical foundation for the development of more effective, durable nanotherapeutic platforms and innovative immunotherapeutic strategies.

From an engineering perspective, neutrophil phagocytosis provides two opposite but complementary design directions. On one hand, biomaterials may be engineered to enhance neutrophil recognition and uptake, thereby improving pathogen clearance or enabling neutrophil-mediated delivery to inflammatory sites. On the other hand, long-circulating nanotherapeutics may need to avoid premature neutrophil uptake through surface shielding or biomimetic cloaking. The major challenge is to control when neutrophil phagocytosis should be promoted and when it should be avoided. Excessive activation of phagocytic neutrophils may aggravate inflammation, whereas excessive immune evasion may reduce host defense or alter biodistribution. Therefore, phagocytosis-oriented engineering should be guided by the disease stage, target tissue and desired therapeutic route.

### Respiratory burst and ROS-related therapeutic modulation

Oxygen-dependent killing represents one of the core mechanisms by which neutrophils eliminate pathogens, primarily through the process of the respiratory burst that generates a spectrum of ROS. Upon activation, the NADPH oxidase complex assembles on the phagosomal or plasma membrane, catalyzing the reduction of molecular oxygen into superoxide anion (O_2_^−^), which initiates a cascade of ROS production. Superoxide is rapidly dismutated into hydrogen peroxide (H_2_O_2_), which, in the presence of MPO, is further converted into potent oxidants such as hypochlorous acid (HOCl). These reactive species inflict extensive damage on microbial membranes, DNA and proteins. In addition, superoxide can react with nitric oxide (NO), often elevated at sites of inflammation, to form the highly oxidative peroxynitrite (ONOO^−^), further amplifying the microbicidal response [112–115]. Although ROS should not be regarded as a homogeneous entity, collectively they establish a highly toxic microenvironment within the phagosome, crucial for efficient pathogen clearance.

Distinct from macrophages, neutrophil phagosomes maintain a neutral to mildly alkaline pH, an environment favorable for the activation of serine proteases such as NE and CG, which act synergistically with ROS to exert potent antimicrobial effects. Beyond their direct microbicidal role, ROS also participate in signaling regulation. Although once considered excessively nonspecific due to their high reactivity, ROS can achieve target specificity through cellular redox buffering systems and restricted local diffusion, thereby modulating enzymes such as phosphatases and proteases and influencing processes including inflammation and apoptosis. Moreover, ion fluxes triggered by ROS generation may contribute to bacterial killing through the release and activation of proteases [116–118]. Thus, oxygen-dependent killing not only constitutes a central mechanism for pathogen clearance by neutrophils but also plays critical roles in signaling, inflammatory regulation and host defense, underscoring its multifaceted functions in innate immunity.

Building on the unique microenvironment shaped by oxygen-dependent mechanisms, Wang *et al.* developed neutrophil membrane-coated nanoparticles loaded with polydopamine to effectively adsorb and neutralize excess neutrophil-associated inflammatory factors at sites of injury. This system scavenged reactive oxygen and nitrogen species, markedly reduced neutrophil infiltration in damaged spinal cord tissue, remodeled the local inflammatory and oxidative stress microenvironment and ultimately promoted neural regeneration and functional recovery [73]. Similarly, neutrophil membrane-derived nanovesicles have been engineered for ROS-responsive release of ranibizumab, thereby inhibiting vascular endothelial growth factor (VEGF) signaling and blocking endothelial cell activation and proliferation [74]. In ischemic brain tissue, ROS-sensitive nanocarriers enabled controlled release of FTY720, increasing local drug delivery by 15.2-fold while significantly reducing cardiotoxicity and infection risk [94]. In another innovative approach, platinum nanoclusters (PtNCs) were anchored onto neutrophil membranes, endowing them with multi-enzyme catalytic activity, ROS-responsive drug release and self-propulsion capacity. These ‘metal-armored’ neutrophil micromotors (Neumotors) efficiently traversed the BBB, accumulated in inflamed brain regions, scavenged excessive free radicals, alleviated neuroinflammation and BBB disruption and significantly improved cognitive and memory functions in mice. This strategy exemplifies the integration of immune targeting with nanocatalytic therapy, offering a novel paradigm for the treatment of neuroinflammatory disorders [119].

ROS-related neutrophil engineering also requires careful balance. ROS-responsive materials, antioxidant systems and neutrophil-mimetic nanoplatforms can reduce oxidative injury and improve local therapeutic release in inflamed tissues. However, complete or nonspecific suppression of ROS may impair antimicrobial defense and redox-dependent repair signaling. In addition, ROS levels vary markedly across tissues, disease stages and injury types, making it difficult to define a universal therapeutic window. Therefore, ROS-modulating biomaterials should ideally provide localized and self-limited regulation rather than systemic or sustained antioxidant effects.

### Oxygen-independent antimicrobial and immunoregulatory mechanisms

The oxygen-independent microbicidal activity of neutrophils relies on their abundant intracellular repertoire of peptides and proteins with direct or indirect antimicrobial functions, forming a crucial arsenal against microbial invasion [150, 151]. These antimicrobial factors can be broadly divided into three categories: cationic antimicrobial peptides/proteins, hydrolytic enzymes and metal-chelating proteins. Cationic antimicrobial peptides such as α-defensins and LL-37 disrupt microbial membranes through electrostatic interactions with negatively charged bacterial surfaces and may also interfere with essential processes including DNA replication, protein synthesis and energy metabolism. Notably, α-defensins inhibit bacterial cell wall synthesis even at low concentrations, suggesting they play a significant role under physiological conditions. LL-37 not only exerts direct antimicrobial effects but also enhances dendritic cell recognition of DNA, thereby contributing to immunomodulation [120–122]. Neutrophil granules are also enriched with hydrolytic enzymes. Lysozyme, for instance, degrades bacterial cell walls, although its antimicrobial function is not entirely dependent on enzymatic activity. Members of the serine protease family, including NE, CG and proteinase 3 (PR3), are activated under specific conditions, enabling them to degrade bacterial virulence factors and participate in inflammatory regulation. Importantly, mutations in NE in humans have been linked to neutropenia and heightened susceptibility to infection, underscoring its essential role in host defense [123–125].

The third category of antimicrobial mechanisms involves metal-chelating proteins, which deprive pathogens of essential metal ions and thereby establish a nutritional immunity barrier. Lactoferrin, for example, binds iron with high affinity, restricting its availability for bacterial growth, while calprotectin chelates zinc and manganese ions, further inhibiting microbial metabolism [126, 127]. Although these molecules exhibit potent antimicrobial activity *in vitro*, their *in vivo* mechanisms remain difficult to dissect due to extensive redundancy and cooperative interactions. Gene knockout models often display only mild or negligible phenotypes, and interspecies differences in the antimicrobial protein repertoire of neutrophils further complicate mechanistic interpretation [152, 153]. Overall, the oxygen-independent killing pathways of neutrophils form a multilayered, multi-molecule cooperative defense system, complementing ROS-mediated bactericidal mechanisms and ensuring host protection under hypoxic conditions or in the setting of impaired ROS generation.

Neutrophil effector functions provide important mechanistic bases for engineering the regenerative microenvironment. Phagocytic clearance, ROS-dependent responses and oxygen-independent antimicrobial mechanisms can be harnessed to support debris removal, antimicrobial defense, immune modulation and targeted delivery [27, 140]. However, these functions are intrinsically double-edged. Excessive phagocytosis-associated activation, uncontrolled oxidative stress or dysregulated protease release may damage host tissues and delay repair. Therefore, effector function-based biomaterials should be designed not simply to amplify neutrophil activity, but to regulate its intensity, localization, duration and compatibility with tissue reconstruction.

Although NET formation is also a neutrophil effector mechanism, it differs from phagocytosis, degranulation and soluble mediator release in both biological form and translational implications. NETs are extracellular DNA-based structures decorated with histones and granule-derived proteins, allowing neutrophils to influence the injured microenvironment beyond cell-intrinsic killing. Their effects are highly context dependent. Controlled NET formation may contribute to pathogen containment, hemostasis and local matrix-like support, whereas excessive or insufficiently cleared NETs can amplify inflammation, induce cytotoxicity, promote thrombosis, aggravate fibrosis and delay regeneration [154, 155]. In addition, NETs have inspired a distinct group of biomaterial strategies, including NET-degrading systems, NET-regulating materials and NET-mimetic platforms. For these reasons, NETs are discussed separately in the following section as a specialized effector mechanism with particular relevance to tissue repair, regeneration and biomaterial engineering.

## Neutrophil extracellular traps: from pathophysiology to regenerative engineering

NETs are a functionally distinctive and currently highly studied effector mechanism of neutrophils, exerting broad and complex influences during tissue injury and repair. As web-like structures composed of chromatin and granule-associated proteins, NETs not only contribute to pathogen trapping and immune defense but also mediate inflammatory amplification, tissue damage and microenvironmental regulation [45, 97]. Owing to their unique formation process and markedly context-dependent biological effects, NETs have become one of the most representative and intensively investigated functional modules in neutrophil biology. Meanwhile, biomimetic designs and targeted intervention strategies developed based on the structural and functional characteristics of NETs are also emerging as important directions in translational research [156, 157]. This section therefore summarizes the formation, regulation and major biological functions of NETs, and further outlines therapeutic strategies developed on the basis of NET biology.

### Formation, regulation and functional roles of NETs

NETs are DNA-protein complexes released by neutrophils in response to various inflammatory and immune stimuli, a phenomenon first described by Brinkmann *et al.* in 2004. These web-like structures are composed of decondensed chromatin fibers decorated with antimicrobial molecules such as histones, MPO and NE. At sites of infection, NETs entrap and kill bacteria, fungi and viruses, thereby preventing pathogen dissemination and serving as an essential component of innate immune defense [154, 158, 159]. Recent studies have further revealed that NETs not only contribute to antimicrobial responses but also play broad roles in diverse pathological processes, including inflammatory diseases, autoimmunity, cancer and thrombosis. Thus, NETs possess a dual nature, while protective in host defense, aberrant or excessive NET activation can disrupt immune homeostasis and cause collateral tissue damage [160].

Under physiological conditions, NETs act as 3D mesh-like barriers that effectively restrict the dissemination of pathogens while their associated antimicrobial proteins directly kill microbes, thereby providing a temporal window for immune clearance [155, 161]. However, when NET formation becomes dysregulated or their clearance is impaired, NETs can transform into pro-inflammatory and pathogenic factors. Histones and proteases released from NETs can directly injure epithelial and endothelial cells, triggering inflammatory cascades and leading to tissue necrosis. Their extensive web-like structures may also entrap tumor or infectious antigens, interfering with their recognition and processing by antigen-presenting cells, thereby suppressing T cell activation and the establishment of immune memory. In addition, NETs can stimulate macrophages to secrete immunosuppressive cytokines such as IL-10 and TGF-β, while promoting Treg cell expansion. These processes foster an immunosuppressive microenvironment that contributes to chronic inflammation and immune evasion [162–164].

The formation of NETs, known as NETosis, represents a distinct form of programmed cell death that differs from apoptosis and necrosis and is characterized by high spatiotemporal specificity [165, 166]. This process is regulated by multiple signaling pathways, with several critical steps. Among them, peptidylarginine deiminase 4 (PAD4)-mediated histone citrullination facilitates chromatin decondensation and nuclear envelope breakdown, while NADPH oxidase-driven ROS generation initiates downstream signaling cascades. In addition, inflammatory pathways such as PKC and MAPK are also involved in modulating NET release. A wide range of stimuli, including bacteria, fungi, viruses, tumor cells, cytokines (IL-8, TNF-α) and immune complexes, can induce NET formation on different temporal scales, enabling NETs to exert diverse and context-dependent functions across infectious, sterile inflammatory and tumor microenvironments [167–170].

### NET-targeted interventions and NET-inspired biomaterial strategies

With the growing understanding of NET biology, targeted interventions and biomimetic engineering have emerged as some of the most dynamic areas in neutrophil research. In intraventricular hemorrhage, neutrophils accumulate extensively in meningeal lymphatic vessels (mLVs) and release NETs, leading to lymphatic endothelial cell (LEC) injury and lymphatic thrombosis. NET-induced upregulation of CX3CR1 further promotes neutrophil recruitment and NET formation, creating a vicious cycle that exacerbates hydrocephalus and neurological dysfunction. Degradation of NETs or genetic ablation of CX3CR1 significantly improved mLV drainage, alleviated hydrocephalus and promoted functional recovery [171]. Similarly, neddylation has been identified as a key regulatory bridge in NET-mediated pathology following traumatic brain injury, representing a potential therapeutic target for preventing or mitigating brain damage [156]. In periodontitis, extracellular histones released from NETs have been identified as central pathogenic mediators. NETs accumulate early in diseased tissues and extracellular histones potently trigger local inflammation and bone destruction. Inhibiting NET formation or neutralizing extracellular histones effectively reduced periodontal bone loss, underscoring the role of NETs as drivers of inflammatory bone resorption. Furthermore, studies have linked NETs to the IL-17/Th17 axis, showing that NETosis directly promotes Th17 responses and establishes a NET-IL-17 inflammatory positive feedback loop, which has been validated in clinical samples. This provides novel insights into how neutrophils contribute to mucosal immune dysregulation [172]. In addition, NETs serve as natural scaffolds that capture platelets and red blood cells, activate coagulation factors and drive immunothrombosis, playing pivotal roles in conditions such as sepsis, atherosclerosis and cancer-associated thrombosis. Advances in single-cell omics and real-time imaging have enabled dynamic tracking of NET formation and spatiotemporal features, offering deeper mechanistic insights into their roles in immune regulation and dysregulation. These discoveries have fueled the exploration of targeted therapeutic strategies, including DNase-mediated degradation of NET structures, PAD4 inhibition to block NET formation and modulation of ROS production to reduce NET release. Each approach has shown potential in the treatment of inflammatory disorders, autoimmune diseases and cancer immunotherapy. Collectively, NETs represent a structural axis of immune regulation that is transitioning from mechanistic discovery to clinical translation and may ultimately serve as a key breakthrough in the development of precision immunotherapies [173–175].

**Figure 5:**
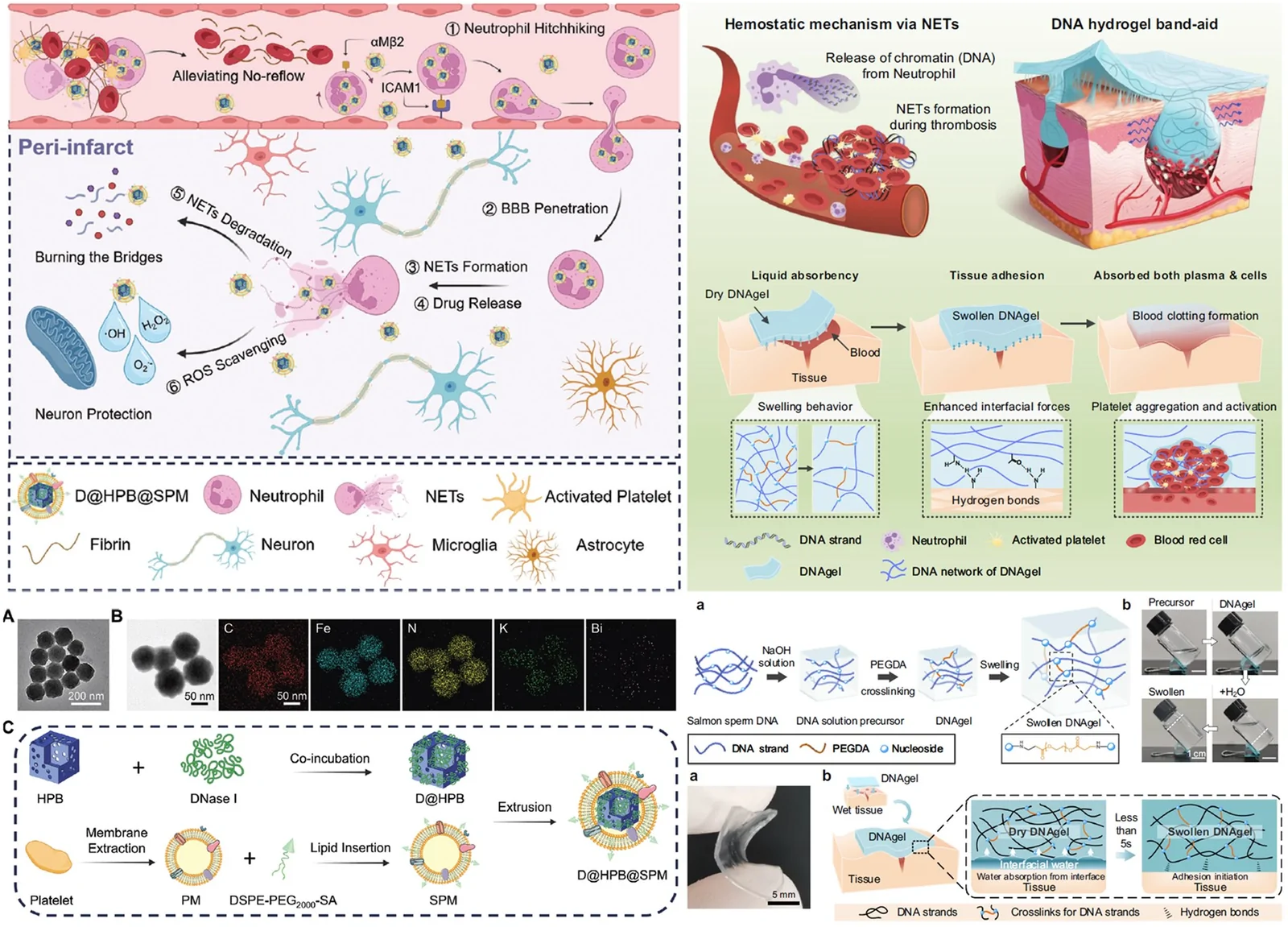
Biomimetic applications harnessing NETs for regenerative regulation. Schematic diagram of the neutrophil-hitchhiking biomimetic nanozymes mediated ischemic stroke therapy. D@HPB@SPM NPs are specifically endocytosed by neutrophils and hitchhike on neutrophils to reach the cerebral ischemic sites. Subsequently, neutrophils are activated and unleash D@HPB@SPM NPs through the production of NETs. D@HPB@SPM NPs not only mitigate neutrophil-induced brain damage by degrading NETs in a manner similar to ‘burning the bridges,’ but also relieve oxidative stress injury by efficiently scavenging ROS (left). Reproduced from Ref. [167] with permission of John Wiley and Sons, *©* 2024; Scheme of DNA hydrogel band-aid for accelerated hemostasis that can fulfill the clinical requirements of stabilizing a profuse bleeding wound. Physiologically, neutrophils at the wound site release extracellular DNA to form traps (NETs) that promote fast and stable thrombosis. Similarly, our form factor conforming DNA hydrogel was able to form NETs-like thrombosis. Besides the conformational advantage, DNAgel is also highly absorptive of blood at the wound edge and highly adhesive to the wetted wound surfaces, thus forming both a physical blockade and biological clot to stop the bleeding (right). Reproduced from Ref. [146] with permission of Springer Nature, *©* 2024.

In terms of biomimetic applications, a ‘neutrophil hitchhiking’ strategy has been employed to construct bioinspired nanozymes (D@HPB@SPM NPs) for precise intervention in ischemic stroke reperfusion injury. This system integrates hollow Prussian blue nanoparticles for ROS scavenging, DNase I for NET degradation and platelet membrane coating to confer inflammation and vascular injury targeting. After internalization by neutrophils, the nanoparticles leverage their innate inflammatory homing capacity to rapidly traverse the blood–brain barrier and release their cargo at lesion sites. This dual-action ‘bridge-burning’ strategy simultaneously clears NETs and eliminates ROS, thereby significantly attenuating neutrophil-mediated inflammation and oxidative stress, offering an innovative biomimetic approach for precision stroke therapy (Figure 5, left) [176]. Similarly, Song *et al.* developed a DNA hydrogel (DNAgel) patch inspired by the structural and pro-coagulant features of NETs. Constructed from natural nucleic acids, DNAgel exhibits rapid water absorption and swelling, instant tissue adhesion and excellent mechanical properties, enabling it to fill tissue cavities and provide compression hemostasis to ruptured vessels. Its highly NET-mimetic network structure effectively promotes erythrocyte adhesion and aggregation, activates platelets and triggers the coagulation cascade. In rat trauma models, DNAgel outperformed commercial gelatin sponges by significantly reducing blood loss, while in full-thickness skin incision models it accelerated wound healing. These findings highlight the translational potential of neutrophil-inspired biomimetic strategies for managing deep, non-compressible hemorrhage (Figure 5, right) [157].

These NET-oriented strategies illustrate two different engineering logics. NET-targeted approaches, such as DNase-loaded systems, NETosis-inhibiting platforms and ROS/NET dual-regulating nanomaterials, aim to reduce pathological NET accumulation and limit secondary inflammation. In contrast, NET-inspired materials use the structural features of extracellular DNA networks to design adhesive, hemostatic or matrix-like biomaterials. Both directions have translational potential, but their safety requirements differ. Excessive NET inhibition may weaken antimicrobial defense or impair early wound stabilization, whereas NET-mimetic materials must avoid unwanted thrombosis, immunogenicity or delayed clearance. Therefore, NET-related biomaterial strategies should be designed according to injury type, infection risk, vascular status and the required duration of local material activity. Overall, NETs constitute a unique functional arm of neutrophils that links host defense with inflammatory injury and tissue repair. Their formation and regulation determine whether NETs act in a protective or pathological manner within damaged tissues. From an engineering perspective, NETs can be either therapeutic targets to be degraded or restrained, or structural inspirations for hemostatic and regenerative biomaterials. A deeper understanding of NET biology is therefore essential for developing strategies that limit NET-associated damage while preserving their beneficial functions. Future translation will require careful control of NET modulation intensity, local duration, infection risk, thrombosis risk and material clearance.

## Targeting neutrophil heterogeneity and plasticity in regeneration

Neutrophil heterogeneity has emerged as a key concept for understanding their diverse roles in tissue regeneration. Rather than acting as a uniform cell population, neutrophils comprise distinct subsets with specialized functions, and their phenotypes can be dynamically reshaped by local microenvironmental cues during repair [6, 177]. Recent advances in single-cell profiling, high-dimensional cytometry and related analytical approaches have greatly expanded the identification of regeneration-associated neutrophil subsets and their context-dependent behaviors. This section therefore focuses on the subset-specific roles of neutrophils in regulating repair outcomes, as well as their phenotypic plasticity and fate switching during regeneration.

### Subset-specific roles in tissue repair outcomes

As key members of the innate immune system, neutrophils have recently been recognized not only for their critical role in antimicrobial defense but also as important regulators of tissue repair and regeneration [15]. Through the secretion of VEGF and MMPs, neutrophils actively promote angiogenesis and facilitate the recruitment and modulation of endothelial progenitor cells, thereby accelerating neovascular formation [178, 179]. Importantly, a recent study revealed that neutrophils are not merely ‘fighters’ in immune defense but can also act as ‘barrier builders’ by depositing collagen and other ECM components within barrier tissues, enhancing both mechanical strength and protective function. Matrix-forming neutrophils, regulated by TGF-β signaling, assemble a collagen ‘wall’ around cutaneous wounds to block the entry of molecules and pathogens. Disruption of this matrix phenotype compromises barrier integrity and heightens infection risk, highlighting neutrophils’ dual role in coordinating immune defense and structural reinforcement in tissues such as the skin [7]. In addition, Segal *et al.* identified an immature neutrophil subset (Ly6G^low^CD14^+^CD101^low^) with neuroprotective and pro-regenerative functions. This subset secretes growth-promoting factors including nerve growth factor (NGF) and insulin-like growth factor-1 (IGF-1), thereby enhancing the survival of injured neurons and stimulating axonal regeneration within the CNS. Remarkably, this regenerative neutrophil population demonstrated potent reparative effects in both optic nerve and SCI models [177].

In tissue engineering, appropriately designed scaffold materials not only help prevent neutrophil overactivation-induced tissue damage but also harness their pro-regenerative activities [75, 180]. Supported by material design strategies such as surface chemical modification, mechanical property tuning and nanoscale structural optimization, neutrophils can be induced to release reparative cytokines, including IL-1Ra and Annexin A1. These factors guide macrophage polarization toward an M2 phenotype, thereby creating an immune microenvironment conducive to tissue regeneration [181, 182]. More recently, emerging immune-smart materials have utilized approaches such as liposomal delivery, RNA interference and micro/nanopatterning to precisely regulate neutrophil behavior, further expanding their translational potential in regenerative medicine. Thus, neutrophils are not merely initiators of inflammation but can also serve as facilitators of regeneration, with their constructive interactions with biomaterials representing a new frontier that integrates tissue engineering with immune modulation [183]. Cai *et al.* further demonstrated that neutrophils are the predominant source of EVs during the early phase of bone defect repair, contributing over 80% of total vesicles. These neutrophil-derived exosomes (PMN-Exos) play a central role in angiogenesis. To exploit this mechanism, the authors developed a phosphatidylserine-modified chitosan hydrogel scaffold capable of efficiently capturing PMN-Exos, which significantly enhanced endothelial progenitor cell proliferation and neovascularization. Mechanistically, PMN-Exos were enriched in miR-455-3p, which targeted and suppressed Smad4 expression in endothelial progenitor cells, thereby augmenting angiogenic capacity. This strategy provides a novel approach to accelerate bone regeneration by leveraging neutrophil-mediated exosomal signaling [184].

### Phenotypic plasticity and fate transition during regeneration

**Figure 6:**
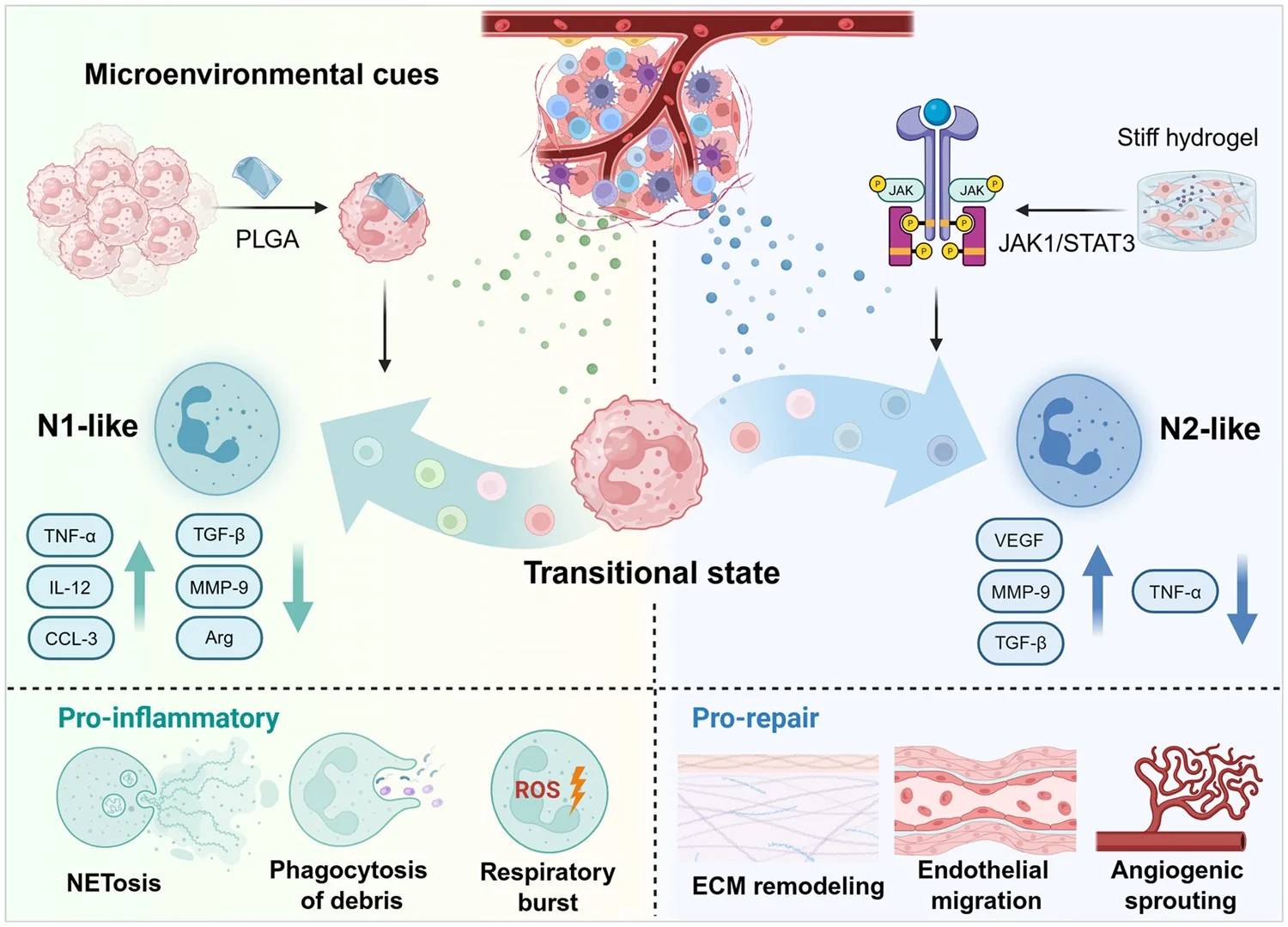
Neutrophil heterogeneity and its regulatory mechanisms in tissue repair and regeneration. Neutrophils exhibit substantial heterogeneity and phenotypic plasticity in response to tissue-specific inflammatory and reparative cues. Different neutrophil states can influence inflammatory resolution, macrophage polarization, angiogenesis, ECM remodeling and tissue reconstruction. Biomaterials can be engineered as immune-instructive platforms to regulate neutrophil recruitment, phenotype switching, cytokine release, EV signaling and interactions with other immune or stromal cells, thereby guiding neutrophil responses toward repair-supportive outcomes. Created with BioRender.com.

As a critical component of the innate immune system, neutrophils have recently drawn increasing attention due to their remarkable heterogeneity and plasticity [185, 186]. Studies demonstrate that within diverse tissue microenvironments, neutrophils can dynamically shift their functional states in response to external cues, participating in a wide spectrum of physiological and pathological processes ranging from inflammation to tissue repair and tumor immunity [19]. Particularly in disease settings, neutrophil subset conversion and functional reprogramming are thought to exert profound effects on local immune states and tissue homeostasis [187]. In oncology, neutrophils are often described within the N1/N2 framework, where N1 generally denotes a more pro-inflammatory and antitumor phenotype, whereas N2 is more commonly associated with protumor, immunosuppressive and tissue-remodeling functions. N1-like neutrophils are typically characterized by elevated expression of inflammatory mediators, including IFN-γ, TNF-α, IL-1β and IL-6, together with enhanced ROS production and NETosis activity, while N2-like neutrophils may arise under signals such as TGF-β and G-CSF (Figure 6) [188–190]. Importantly, increasing evidence suggests that neutrophils are more accurately represented as a continuum of context-dependent functional states rather than as two fixed and mutually exclusive subsets. Transcriptomic studies likewise indicate substantial phenotypic and functional diversity within neutrophil populations, with their transitional states and biological outputs being continuously shaped by microenvironmental cues. Accordingly, during acute and chronic inflammation as well as tissue repair, changes in neutrophil functional composition are more likely to reflect dynamic state transitions along a heterogeneous continuum, rather than a simple shift in the relative abundance of two discrete populations [191, 192]. Leveraging this plasticity, Liu *et al.* designed a bioactive microporous hydrogel scaffold based on natural polysaccharides, with promising translational potential. By incorporating N2-conditioned medium into a microporous GelMA hydrogel, they generated an N2 hydrogel scaffold with superior mechanical properties. Preclinical evaluations demonstrated that this scaffold promoted the expression of anti-inflammatory and vascular maturation genes, and significantly enhanced angiogenesis through improved endothelial cell migration, tubular network formation and anastomosis. The N2 hydrogel scaffold exhibited outstanding therapeutic efficacy in models of ischemic disease [193].

Further studies suggest that the physical and biochemical properties of scaffolds do not merely serve as passive carriers, but can directly shape neutrophil phenotype and functional state. Jiang *et al.* systematically demonstrated that matrix stiffness markedly regulates the polarization of bone marrow-derived neutrophils, with stiffer matrices promoting their transition toward an N2-like, anti-inflammatory and tissue repair-associated phenotype, whereas softer matrices were more likely to maintain or induce pro-inflammatory features. Mechanistically, this effect was closely associated with activation of the JAK1/STAT3 signaling pathway [194]. Meanwhile, Witt *et al.* showed that neutrophils can actively sense environmental stiffness and dynamically adjust their traction force output, while their activation state further influences traction magnitude, force distribution patterns and migratory behavior, suggesting that mechanical cues not only regulate cell migration but may also be deeply involved in shaping neutrophil inflammatory functions [195]. Collectively, key scaffold features, including stiffness, ligand presentation and degradability, can influence inflammatory progression and repair outcomes by regulating neutrophil recruitment, polarization and mechanosensitive responses. This further highlights the remarkable plasticity of neutrophils, positioning them as key regulators not only of inflammatory control but also of the transition from inflammation to regeneration [6].

Despite their critical role in immune regulation, the application of neutrophils in adoptive cell therapy remains relatively underdeveloped, particularly when compared with more established therapeutic cell types such as T cells and macrophages [60, 70, 147]. This lag is partly due to the strong microenvironmental dependency of neutrophil function and the lack of stable, controllable induction methods. In addition, their robust phagocytic capacity confers a high tendency to internalize nanodrug carriers, making it difficult to maintain a controlled and durable phenotype [196, 197]. Conventional ‘cell backpack’ strategies often rely on nanoparticle-mediated cytokine delivery to induce and sustain specific phenotypes, such as maintaining macrophages in an M1 state. However, in neutrophils, such approaches are limited in efficacy and often compromised by rapid uptake and degradation. Recently, an innovative neutrophil engineering strategy was developed in which PLGA micropatches were attached to the neutrophil surface as drug-free ‘backpacks.’ These structural patches reprogrammed neutrophils toward an antitumor N1 phenotype through physical modulation, providing them with stable and persistent activity. Unlike traditional drug- or cytokine-dependent backpack systems, this approach activates neutrophils solely via cytoskeletal remodeling, leading to marked upregulation of antitumor effectors such as TNF-α and MPO, while further stimulating NK cells and T cells to amplify systemic immune responses. This strategy not only expands the scope of neutrophil-based adoptive cell therapy but also exemplifies a novel concept of directing immune cell fate through purely structural design. It demonstrates advantages in safety, controllability and translational potential. Nonetheless, its application in tissue repair contexts remains largely unexplored [20].

Overall, neutrophil heterogeneity provides an important framework for reinterpreting their roles in tissue regeneration. The identification of repair-associated subsets and the recognition of phenotype switching under microenvironmental regulation together indicate that neutrophils participate in regeneration in a far more specialized and dynamic manner than previously appreciated. These advances not only deepen the mechanistic understanding of neutrophil biology in repair but also offer new opportunities for subset-specific targeting and phenotype-guided therapeutic modulation.

## Neutrophil-centered cellular crosstalk in regenerative niches

Beyond their intrinsic effector functions, neutrophils actively shape tissue regeneration through dynamic crosstalk with immune and structural cells in regenerative niches. Their interactions with monocytes and macrophages, regulation of lymphocyte responses and communication with epithelial cells together influence inflammatory resolution, barrier restoration and tissue remodeling [198]. Increasing evidence suggests that these intercellular interactions are not only central to the regenerative functions of neutrophils but also provide an important basis for biomimetic and engineered therapeutic strategies [102]. This section therefore summarizes how neutrophils orchestrate cellular crosstalk in regenerative niches and highlights the corresponding translational potential.

### Interactions with monocytes and macrophages

During tissue infection or injury, neutrophils and their close lineage relatives, monocytes and macrophages, coordinate their activities in a temporally ordered manner, leading to sequential recruitment of these cell populations [28]. Macrophages and patrolling monocytes are among the earliest cells to sense PAMPs or DAMPs, and they initiate the recruitment of large numbers of neutrophils to sites of inflammation. Following the influx of neutrophils, monocytes arrive shortly thereafter, suggesting a causal relationship underpinning this temporal sequence [2, 60, 199]. Indeed, neutrophils actively promote monocyte recruitment through multiple mechanisms. They express classical monocyte-attracting chemokines such as CCL2, CCL3, CCL20 and CCL19. In addition, neutrophils release granule-derived proteins, including LL-37, heparin-binding protein (HBP/CAP37) and CG, which further drive monocyte extravasation *in vivo* [200]. Monocyte recruitment is also indirectly influenced by neutrophils, which upregulate endothelial adhesion molecules, increase transendothelial permeability, stimulate chemokine production by other cell types, and regulate chemokine activity through proteolytic processing [201, 202]. Beyond recruitment, neutrophils can modulate cytokine production by monocytes and macrophages, directly enhancing their microbicidal activity. The cyclical nature of these interactions becomes particularly evident during inflammation resolution: monocytes recruited by neutrophils and subsequently differentiated into macrophages suppress further neutrophil chemotaxis and ensure proper clearance of apoptotic neutrophil remnants [82]. Calcitonin gene-related peptide (CGRP)-positive sensory neurons also play a regulatory role by acting on RAMP1 expressed on neutrophils and macrophages. This signaling pathway suppresses immune cell accumulation, accelerates immune cell apoptosis, enhances macrophage efferocytosis and promotes macrophage polarization toward a pro-repair phenotype, thereby accelerating tissue healing. These functions of CGRP are mediated by thrombospondin-1 (TSP-1), highlighting a neuroimmune axis that supports tissue repair. Therapeutically, CGRP can be harnessed to correct neuroimmune dysregulation and effectively address impaired wound healing in conditions such as diabetes [15]. Addressing the challenge of excessive neutrophil infiltration and inflammatory imbalance in SCI, Chen *et al.* developed a biomimetic neural regeneration scaffold (NM-MS) that combines the multitarget ‘inflammatory decoy’ effect of neutrophil membrane vesicles (NMVs) with microsol electrospinning and cell membrane coating techniques. The scaffold not only neutralizes inflammatory signals and suppresses neutrophil recruitment during the acute phase but also, once internalized by macrophages, promotes their polarization toward an anti-inflammatory M2 phenotype. In the chronic phase, sustained release of BDNF further enhances neural regeneration. This spatiotemporally precise regulation of inflammation and neural repair provides a novel strategy for reconstructing the neutrophil–macrophage axis in regenerative medicine [203].

### Coordination of lymphocyte responses

A surprising finding in recent years is the extensive crosstalk between cells positioned at opposite ends of the immune spectrum. Neutrophils and T cells, once thought to reside in separate functional compartments, in fact shape and influence each other’s activity [204]. Neutrophils can modulate T-cell responses indirectly via dendritic cells (DCs) as well as directly. For example, neutrophil-derived IL-12 may be critical for Th1 differentiation, while neutrophils also produce multiple T-cell attracting chemokines and B-cell development and maturation factors [205, 206]. Cytokine communication between these cell types is bidirectional. T cells secrete IFN-γ, which prolongs neutrophil survival, induces gene expression and enhances their phagocytic activity. Moreover, IL-17 secreted by the Th17 subset has emerged as a key cytokine regulating neutrophil dynamics. Acting through epithelial, endothelial and stromal cells, IL-17 promotes the expression of IL-8, G-CSF and TNF-α. Collectively, these Th17-associated cytokines stimulate granulopoiesis, drive neutrophil recruitment and extend neutrophil lifespan [207, 208].

Proposed mechanisms for neutrophil-mediated suppression of T cells include: (i) the release of arginase, which depletes extracellular L-arginine and thereby inhibits T-cell activity *in vitro*; and (ii) H_2_O_2_-mediated suppression, as demonstrated in cancer models [209, 210]. However, direct *in vivo* evidence for these interactions remains limited. Interestingly, neutrophils can also influence CD8^+^ T-cell responses through cross-presentation of exogenous antigens. Beauvillain *et al.* showed, using mice whose professional antigen-presenting cells lacked functional MHC class I molecules, that antigen-pulsed neutrophils were sufficient to induce cytotoxic T-cell differentiation. This finding suggests that neutrophils possess antigen-presenting cell-like properties. Under inflammatory conditions, neutrophils also appear capable of expressing MHC class II molecules and costimulatory molecules, and of presenting antigens to CD4^+^ T cells *in vitro*. Nevertheless, the functional significance of this phenomenon for protective immunity remains uncertain, particularly given evidence that in mice, neutrophil migration to lymph nodes can exert negative effects on CD4^+^ T-cell responses [211]. In humans, the capacity of neutrophils to express MHC class II varies considerably among individuals, suggesting that their ability to activate T cells may also differ. Such interindividual variation may have implications for susceptibility to autoimmune disease. Overall, neutrophil regulation of adaptive immunity is highly complex, and current insights remain only preliminary.

### Neutrophil-epithelial cell communication and barrier restoration

The epithelial barriers of the skin and intestine play pivotal roles in defending against external environmental insults. Importantly, these barriers are capable of rapid restoration following injury, a process that depends on finely coordinated repair mechanisms between epithelial tissues and the immune system [212]. After wounding, dynamic interactions between epithelial cells and both resident and infiltrating innate immune cells, such as neutrophils and monocytes/macrophages, jointly drive barrier reconstruction and the re-establishment of tissue homeostasis. Disruption of this coordination can result in chronic non-healing wounds, significantly increasing morbidity and mortality associated with related diseases. Therefore, elucidating the signaling crosstalk between epithelial cells and immune cells during wound repair is of critical importance for developing therapeutic strategies to treat chronic injuries arising from conditions such as inflammatory bowel disease and diabetes [213–215].

Overall, neutrophils are now recognized not only as effector cells but also as central regulators of intercellular communication in regenerative niches. Their coordinated interactions with monocytes and macrophages, lymphocytes and epithelial cells shape the transition from inflammation to repair and barrier re-establishment. This expanded perspective on neutrophil function not only deepens mechanistic insight into tissue regeneration but also opens new avenues for biomimetic design and therapeutic strategies targeting cellular crosstalk.

## Clinical progress and translational challenges

With the expanding understanding of neutrophil biology in tissue injury and regeneration, increasing efforts have been directed toward translating neutrophil-based strategies from experimental research into preclinical and clinical settings. Emerging evidence from pathway and mechanism-oriented studies has begun to support the therapeutic potential of targeting neutrophil recruitment, activation, effector functions and derived biomimetic systems. At the same time, the complexity of neutrophil biology also presents substantial barriers to clinical application [55, 203]. This section therefore summarizes current preclinical and clinical progress in neutrophil-based therapies and further discusses the major translational challenges that must be addressed for their safe, standardized and scalable therapeutic use.

### Preclinical and clinical trials

To capture the current state of translation in a more structured manner, this section summarizes representative preclinical and clinical studies of neutrophil-related therapies in a table organized by signaling pathways and mechanisms of action. This framework is intended to illustrate how distinct therapeutic strategies converge on neutrophil recruitment, activation, effector programs and downstream inflammatory or reparative processes across different disease settings. It also allows clearer comparison of mechanistic targets and translational trajectories within the field. Where available, we further include key information from ongoing clinical trials, including NCT number, trial status, phase, study objectives and any reported preliminary outcomes. Together, these data provide a concise overview of the current clinical landscape and help define the opportunities and limitations that shape the translational development of neutrophil-based interventions (Table 3).

**Table 3:** Preclinical and clinical trials.

ID	Mechanism/Pathway	Target	Drug	Conditions	Enrollment	Strategy	Phase/Status
NCT05453695	NETs/cfDNA Axis	NETs/cfDNA	Dornase Alfa	Sepsis	36	Systemic NET dismantling	Phase 1/Recruiting
NCT05203224	NETs/cfDNA Axis	NETs/cfDNA	Dornase Alfa	Ischemic Stroke	300	NET-rich thrombus disruption	Phase 2/Recruiting
NCT01952470	NETs/cfDNA Axis	NETs/cfDNA	Dornase Alfa	Lung Transplant Infection	32	Airway eDNA reduction	Phase 2/Completed
NCT03368092	NETs/cfDNA Axis	NETs/cfDNA	Dornase Alfa	ARDS	500	NET/eDNA degradation	Phase 3/Recruiting
NCT03161431	Mobilization, Recruitment and Chemotaxis	CXCR 1/2	SX-682	Melanoma Stage III/IV	77	MDSC trafficking blockade	Phase 1/Active, not recruiting
NCT01890148	Mobilization, Recruitment and Chemotaxis	CXCR2	AZD5069	Asthma	13	Reduction of airway neutrophil recruitment	Phase1/Completed
NCT01704495	Mobilization, Recruitment and Chemotaxis	CXCR2	AZD5069	Asthma	1147	CXCR2-mediated neutrophil recruitment inhibition	Phase 2/Completed
NCT00967785	Mobilization, Recruitment and Chemotaxis	CXCR4	Plerixafor (Mozobil)	WHIM Syndrome	20	CXCR4 antagonism and leukocyte mobilization	Phase 1/2/Recruiting
NCT07118930	Neutrophil Elastase Axis	Neutrophil elastase	Sivelestat Sodium	CAD	62	Neutrophil elastase inhibition	Phase 2/Recruiting
NCT02669251	Neutrophil Elastase Axis	Neutrophil elastase	Alvelestat	Bronchiolitis Obliterans Syndrome	14	Neutrophil elastase suppression	Phase 1/2/Active, not recruiting
NCT02597101	Neutrophil Elastase Axis	Neutrophil elastase	AZD9668	Type 2 Diabetes Mellitus/Insulin Resistance	14	Neutrophil-driven inflammation reduction	Phase 2/Completed
NCT03218917	Upstream Protease Maturation	DPP-1 (Cathepsin C)	Brensocatib (INS1007)	Non-CF Bronchiectasis	256	Reduced activation of neutrophil serine proteases	Phase 2/Completed
NCT04594369	Upstream Protease Maturation	DPP-1 (Cathepsin C)	Brensocatib (INS1007)	Non-CF Bronchiectasis	1767	DPP-1 inhibition and exacerbation reduction	Phase 3/Completed

The data source: https://clinicaltrials.gov/.

### Translational challenges of clinical therapies

Although neutrophil-related therapies hold considerable promise in inflammation control, infection clearance, tissue repair and targeted delivery, their clinical translation remains more challenging than that of many established cell-based platforms [70, 134]. A major difficulty arises from the intrinsic biology of neutrophils. They are highly reactive, short-lived effector cells, and the same mechanisms that support host defense, including degranulation, ROS production, NET formation, protease release and inflammatory mediator secretion, may also cause unintended tissue injury or off-target inflammatory toxicity if not precisely controlled [216, 217]. Therefore, the therapeutic goal should not be broad activation or suppression of neutrophils, but context-specific regulation of their timing, intensity, localization and resolution.

Neutrophil heterogeneity and functional plasticity represent another important translational barrier. Neutrophil phenotypes and functions vary according to tissue type, injury stage, disease condition, inflammatory intensity, ECM cues and interactions with macrophages, endothelial cells, stromal cells and progenitor cells [19]. As a result, a strategy that promotes repair in one context may aggravate inflammation, fibrosis, thrombosis or tissue damage in another. Future preclinical studies should therefore define not only whether a neutrophil-targeted strategy is effective but also when, where and under which inflammatory conditions it is beneficial. This will require more standardized injury models, time-resolved analysis of neutrophil states and clearer links between neutrophil phenotypes and functional repair outcomes [218, 219].

Safety evaluation is particularly important for neutrophil-targeted interventions. Excessive inhibition of neutrophil recruitment, ROS production, protease activity or NET formation may impair antimicrobial defense and delay early tissue clearance. In contrast, excessive stimulation or prolonged retention of neutrophils may amplify inflammation and damage surrounding tissues [154, 170]. For neutrophil-derived vesicles, membrane-coated nanoparticles, granule-inspired components and engineered neutrophil carriers, additional concerns include immunogenicity, off-target accumulation, thrombosis risk, uncontrolled cargo release and long-term biosafety [27]. Therefore, safety assessment should include not only general biocompatibility but also host defense capacity, inflammatory persistence, vascular effects, biodistribution, clearance and repeated-administration risks.

Manufacturing and quality control also remain major obstacles. Native neutrophils, neutrophil membranes, vesicles, granule-derived components and engineered neutrophil formulations are highly sensitive to donor variability, isolation procedures, *ex vivo* manipulation, storage conditions and activation status [20, 220]. These factors may lead to phenotypic instability, functional variability and batch-to-batch inconsistency. In addition, the short lifespan, poor *ex vivo* stability and limited expandability of neutrophils impose substantial constraints on large-scale production, transport, storage and clinical-grade manufacturing. Compared with T cell- or stem cell-based therapies, neutrophil-related platforms still lack mature manufacturing workflows, standardized potency assays and clearly defined release criteria.

From a regulatory perspective, neutrophil-related products may fall into different categories depending on whether they are based on living cells, cell-derived vesicles, membrane-coated particles, synthetic biomimetic systems or drug-loaded carriers. Each product type may require different standards for identity, purity, potency, sterility, stability, biodistribution and safety. For living neutrophil-based or neutrophil-associated carrier systems, additional issues include cell viability, activation state, cargo retention, functional persistence and risk of inflammatory dissemination. For biomaterial-based immune modulation, regulatory evaluation should also consider degradation products, local persistence, immune compatibility and potential interference with normal wound defense.

Overall, neutrophil-related regenerative strategies are moving from biological concept toward translational development, but this transition remains incomplete. The field is no longer limited mainly by therapeutic rationale, but by the challenge of converting neutrophil biological complexity into safe, controllable, reproducible and manufacturable interventions. Future progress will depend on integrating mechanistic insight, precise engineering control, standardized manufacturing, robust quality testing and clinically relevant validation. Only through such a translational framework can the therapeutic potential of neutrophil-related strategies be more fully realized in tissue repair and regeneration.

## Conclusion and prospects

Neutrophils have traditionally been viewed as short-lived terminal effectors of acute inflammation, yet this framework is no longer sufficient to explain their roles in tissue injury and regeneration [101]. As discussed throughout this review, neutrophils act far beyond early infiltration, participating in debris clearance, oxidative and non-oxidative effector programs, NET formation, phenotypic diversification and bidirectional communication with immune and structural cells [31]. In our view, the most significant advance in the field is not the identification of any single additional function, but the recognition that neutrophils operate as dynamic and context-responsive regulators within evolving regenerative systems.

A second important conclusion is that neutrophil function in regeneration cannot be interpreted within a static or uniform model. As seen in the limitations of oversimplified M1/M2 or Th1/Th2 paradigms, binary classifications are inadequate to capture the diversity, plasticity and context dependence of neutrophil responses [46]. Their effects are shaped by temporal stage, anatomical location, disease context, ECM cues and intercellular interactions, which together determine whether neutrophils exacerbate injury, promote resolution or support tissue reconstruction [7]. Thus, neutrophil heterogeneity should be regarded not as a peripheral observation, but as a central organizing principle for understanding regenerative immunity.

We further propose that the translational future of neutrophil biology lies not in broadly suppressing or amplifying neutrophil responses, but in directing them with precision. Recent advances in neutrophil-inspired engineering, biomimetic 3D culture systems, membrane-based delivery platforms, cell-mediated *in situ* transport and NET-oriented interventions indicate that the field is entering a more programmable phase [221]. These strategies are particularly relevant for overcoming intrinsic limitations of neutrophils, including their short lifespan, poor expandability and difficulty in maintaining function *ex vivo* [194]. In this context, the next major breakthrough will likely come not simply from identifying additional mechanisms, but from establishing platforms capable of selectively controlling neutrophil behavior across space, time, phenotype and function.

Looking forward, we believe that three transitions will shape the next stage of this field. First, research must move from describing heterogeneity to resolving functional heterogeneity, linking specific subsets and trajectories to defined regenerative outcomes. Second, therapeutic development must advance from proof-of-concept studies to integrated translational platforms that combine mechanistic insight, engineering control and clinical feasibility. Third, neutrophils should increasingly be considered not merely as inflammatory effectors or isolated therapeutic targets, but as programmable hubs within multicellular regenerative systems. If these transitions can be achieved, neutrophil-based strategies may evolve from an emerging concept into a practical platform for tissue repair, regenerative medicine and precision immunoengineering.
